# A computational framework to dissect imputation strategies for single-cell histone modification data

**DOI:** 10.1093/nargab/lqaf192

**Published:** 2025-12-29

**Authors:** Marta Moreno-González, Jeroen de Ridder, Jop Kind, Robin H van der Weide

**Affiliations:** Oncode Institute, Hubrecht Institute—KNAW and University Medical Center Utrecht, 3584 CT, Utrecht, The Netherlands; Department of Molecular Biology, Faculty of Science, Radboud Institute for Molecular Life Sciences, Radboud University, 6525 GA, Nijmegen, The Netherlands; Oncode Institute, Center for Molecular Medicine, UMC Utrecht, 3584 CX, Utrecht, The Netherlands; Oncode Institute, Hubrecht Institute—KNAW and University Medical Center Utrecht, 3584 CT, Utrecht, The Netherlands; Department of Molecular Biology, Faculty of Science, Radboud Institute for Molecular Life Sciences, Radboud University, 6525 GA, Nijmegen, The Netherlands; Oncode Institute, Hubrecht Institute—KNAW and University Medical Center Utrecht, 3584 CT, Utrecht, The Netherlands

## Abstract

Single-cell profiling of histone post-translational modifications (scHPTMs) offers a powerful lens for dissecting epigenetic regulation and cellular identity, yet low read depth and inherent noise in these datasets pose significant analytical challenges. Here, we introduce the first comprehensive computational framework that systematically evaluates imputation strategies on scHPTM data, including methods originally developed for scRNA-seq and scATAC-seq. Leveraging both synthetic and published datasets, we apply novel performance metrics—implemented in a modular R package—to assess signal recovery, enrichment at biologically relevant genomic sites, and preservation of cell-to-cell similarities. Our extensive benchmarking reveals that performance varies markedly by analytical task (e.g. signal denoising, peak detection, and clustering), highlighting that no one-size-fits-all solution exists for these data. By delineating the strengths and limitations of current imputation approaches, this work lays the foundation for the targeted development of next-generation, task-aware algorithms, while providing critical guidance for researchers and developers on the current capabilities and unmet needs in single-cell epigenomics.

## Graphical Abstract

**Graphical Abstract:**
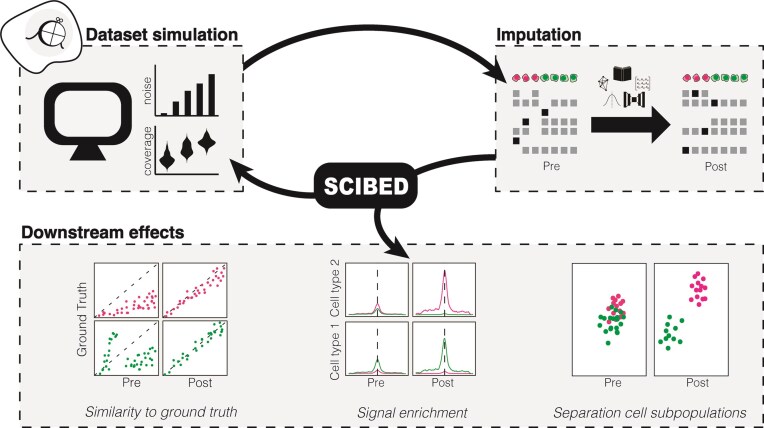

## Introduction

In recent years, significant advances have been made in single-cell histone post-transcriptional modifications (scHPTM) profiling [1–4]. Profiling HPTMs can provide deeper insights into the regulatory states defining cell identity, chromatin accessibility, and gene expression potential. This complements single-cell transcriptomics by focusing on the chromatin context that guides gene expression. Single-cell approaches offer unique insights into several fields, such as tumor heterogeneity in cancer research [5], lineage-tracing studies [6], and early embryogenic development [7].

Despite the promise of scHPTM assays, the data is often sparse and noisy [8, 9]. These methods typically target a specific HPTM via a reader (often in the form of an antibody) and then enzymatically cleave the surrounding DNA [3, 4]. Sparsity arises mainly due to technical limitations related to loss of DNA material (similar to single-cell RNA sequencing (scRNA) and scATAC [10]), suboptimal enzymatic reactions, and antibody quality. This leads to limited coverage per cell, ranging from a hundred to several thousand unique reads per cell [3, 11, 12]. At the same time, noise originates from nonspecific binding and other technical artefacts, which can obscure the biologically relevant (i.e. foreground) signal. These challenges complicate data analysis, making distinguishing true chromatin features and cell subpopulations difficult, potentially leading to misinterpretation if not addressed.

Imputation is a computational approach developed to mitigate data sparsity and noise by estimating or “filling in” missing values. In single-cell genomics, imputation is performed to improve data quality, enhance signal clarity, and bolster cell clustering, all of which contribute to the reliable characterization of cell types and epigenetic landscapes [13]. Various algorithms have been successfully applied to scRNA and single-cell chromatin accessibility (scATAC) data, which face similar challenges [14–16]. scRNA, scATAC, and scHPTM data all rely on counting the number of reads mapping back to specific genomic locations (e.g. genes, open chromatin, or genomic bins) to profile cellular states at a single-cell level. While imputation algorithms are yet to be applied to scHPTM data, we hypothesize that certain imputation methods designed for scRNA or scATAC may also improve scHPTM data quality given the parallels between scHPTM and other single-cell omics.

Single-cell imputation methods build on different computational principles. By examining these diverse methods, we aim to determine which approaches most effectively address scHPTM data challenges:

- Statistical modeling: Some methods leverage assumptions on the data distribution according to technical, biological and noise variability. For this category, we selected SAVER [17] and scImpute [18], two methods developed for scRNA data. SAVER assumes the data has a negative binomial distribution, while scImpute uses a mixture model. scImpute works with some *a priori* information on the number of expected cell subpopulations.
- Data smoothing: These methods try to identify similar cells and aggregate the information across them to impute/denoise the profiles. For this category, we selected KNN-smoothing [19] and MAGIC [15], both methods developed for scRNA data. K-nearest neighbors (KNN)-smoothing uses a simple iterative nearest neighbors approach to identify similar cells and share information across them, while MAGIC uses latent dimensions to determine nearest neighbors before smoothing signal among these.
- Matrix factorization: These methods decompose the observed matrix in a low-dimensional space to remove noise. For this category, we selected ALRA [20] and scOpen [10]. ALRA was developed for scRNA and is based on low-rank matrix factorization, which decomposes a matrix into two products of smaller, simpler matrices. In contrast, scOpen was designed with scATAC data in mind and uses non-negative matrix factorization, a matrix factorization approach that requires all elements in the decomposed matrices to be non-negative.
- Autoencoders: Newer methods leverage autoencoders, a self-supervised learning technique where neural networks learn compressed data representations by minimizing reconstruction error. For this category, we selected DCA [21], SCALE [16], and SCALEX [22]. DCA combines autoencoders with specialized loss functions to learn the true zero-noise data manifold. SCALE combines variational autoencoders with a Gaussian Mixture Model. SCALEX combines variational mixture models with online integration. DCA was designed for scRNA data, SCALE for scATAC data, and SCALEX has settings for both scRNA and scATAC data.
- Topic modeling: Topic modeling is a technique used to identify hidden topics or structures in a collection of documents (or cells) by identifying co-occurrence patterns. cisTopic [23] is designed to identify regulatory topics within scATAC data. While cisTopic was not developed for data imputation, it has been previously adapted for this (*cisTopic-impute*) [10].

**Figure 1.**
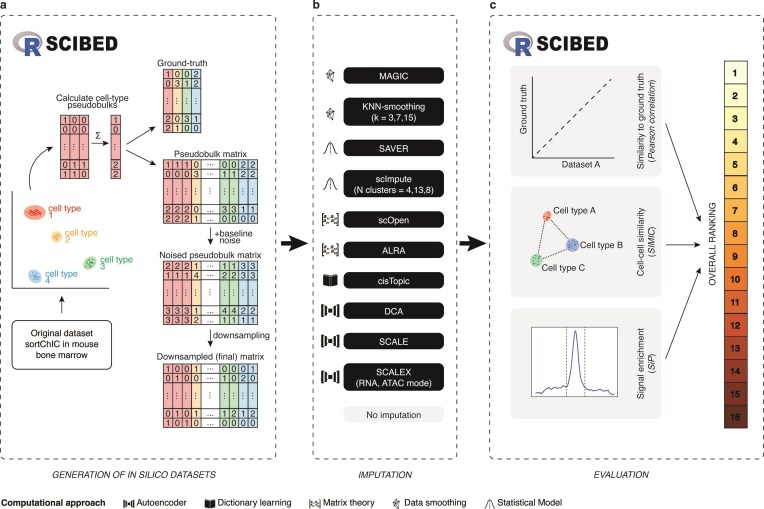
A computational pipeline to benchmark single-cell imputation algorithms on *in silico* epigenomic data. Overview of the computational pipeline. (**A**) *In silico* datasets are generated from an input dataset, with user-specified levels of noise and read depth per cell. (**B**) These *in silico* datasets are used as input for the imputation algorithms tested. (**C**) The output datasets of these algorithms and the dataset pre-imputation were evaluated and ranked according to their correlation to the ground truth dataset, cell–cell similarity scores, and signal at expected genomic regions. The *in silico* dataset generation and evaluation steps are publicly available through SCIBED, and R package for benchmarking single-cell imputation methods in epigenomic data.

In this study, we present a benchmarking framework to evaluate imputation methods for scHPTM data. We applied the aforementioned methods to *in silico* datasets for various histone marks, with controlled levels of sparsity and noise (Fig. 1A and 1B), as well as already published datasets. The resulting imputed datasets were then used to systematically assess algorithm performance in restoring true epigenetic profiles and improving the clustering of cell subpopulations (Fig. 1C). Our evaluation aims to rank these imputation methods for enhancement of scHPTM data quality, while also identifying both the advantages and pitfalls of the different underlying computational principles. This dual focus on benchmarking and methodological insight will support future development of specialized tools for single-cell histone modification analysis. Additionally, we developed an R package, SCIBED (Single-Cell Imputation Benchmarking of Epigenomic Data), which allows users to generate *in silico* scHPTM datasets and evaluate the performance of imputation algorithms on epigenomic data.

## Materials and methods

### SortChIC bone marrow datasets

We used mouse bone marrow sortChiC data for H3K4me1, H3K4me3, H3K9me3, and H3K27me3 from Zeller *et al.* [3] (Supplementary Table S1). Because the public dataset is a combination of two batches, we requested the dataset of the newer batch by personal correspondence.

### scCUT&Tag mouse brain datasets

We downloaded raw mouse brain scCUT&Tag datasets for H3K4me3, H3K27ac, H3K27me3, and H3K36me3 from GEO GSE163532 [4] (Supplementary Table S2). The data were processed as described in the original paper. In order to test the performance of the imputation algorithms, the data was downsampled to ∼1000 read/cell using the *SampleUMI* function of the *Seurat* package [24] prior to imputation.

### *In silico* dataset generation

To systematically benchmark methods with control over noise- and read depth-levels, we generated *in silico* single-cell datasets. These are based on the mouse bone marrow sortChiC H3K4me1, H3K4me3, H3K9me3, and H3K27me3 datasets [3]. We generated *in silico* datasets per modality with 10 different levels of noise and 3 different read depth levels, resulting in 30 datasets per modality. We describe this in more detail below. In addition to these datasets, we also generated scenarios with extremely high levels of noise (>10 times baseline) to test the limits of the algorithms, although these were not considered for the final ranking.

#### Filtering based on read depth and fraction of zero counts

To limit batch effects, we selected on batch (“new”) and transformed the read-count matrices to fragment-count matrices when applicable. The datasets were then filtered according to read depth and fraction of zero counts, as described in the original paper. Cells were filtered for a minimum of 500 reads per cell for the H3K4me1 and H3K4me3 datasets, and 1000 reads per cell for the H3K9me3 and H3K27me3 datasets. We also removed the cells with a fraction of zero counts below two standard deviations from the mean across cells, as cells with a smaller fraction of zero counts are more likely to have unspecific cuts.

#### Generating ground truth pseudobulks

We obtained 14 cell type-specific labels based on the original dataset for the filtered cells and aggregated the counts per cell type to generate pseudobulks. These pseudobulks were used as a ground truth for the evaluation steps. They were also used to generate a pseudobulk count matrix, with as many pseudobulks per cell type as cells in the filtered dataset. This pseudobulk count matrix was used to generate the datasets with different levels of noise and read depths.

#### Adding noise

Noise present in datasets from single-cell epigenomic techniques is often a result of unspecific cuts in the DNA. To reflect this, we expanded the pseudobulk count matrix (*P*) to cover the entire genome in 50 kb bins, and added noise uniformly. The noise added is determined by the average number of counts per bin multiplied by the desired noise level (*l*). For pseudobulk matrix with noise N we write:


(1) \begin{eqnarray*}
N = P + \frac{{\mathop \sum \nolimits_{i = 1}^m \mathop \sum \nolimits_{j = 1}^n {{P}_{ij}}}}{n} \times l
\end{eqnarray*}

We generated datasets with 10 different noise levels, classified as no noise ($l = 0$), low noise levels ($l = 0.25| {0.35} |0.5$ so that the signal-to-noise ratio $ \ge 0$), mid noise levels ($l = 0.75| 1 |1.25$ so that $1.4 \ge $ signal-to-noise ratio $ \ge 0.8$) and high noise levels ($l = 2| {3.5} |5$ so that the signal-to-noise ratio $ \le 0.5$). For H3K4me3, we generated datasets with two additional levels of noise ($l = 10|50$ so that the signal-to-noise ratio $ \le 0.05$) to illustrate scenarios with extremely high levels of noise.

#### Downsampling

In order to accurately reflect the sparsity of single-cell data and introduce cell-to-cell variability, we downsampled the pseudobulk matrix with noise using the *SampleUMI* function of the *Seurat* package [24]. To test the effect of different read depths on the imputation performance, we generated 3 datasets with different read depths (10 000 reads per cell, 1000 reads per cell, and 100 reads per cell) per noise level.

### Imputation methods

All the following imputation methods were run on a high performance computing (HPC) node, providing a maximum of 30 Gb of RAM and 48 h of runtime. In cases where the algorithm requirements exceeded those provided, the relevant algorithms were marked as having a resource error for those specific scenarios.

#### Statistical models

##### SAVER

SAVER [17] (Single-cell Analysis Via Expression Recovery) is an imputation and denoising algorithm developed for scRNA that models gene expression per cell as a negative binomial distribution. The latest version of SAVER was downloaded from https://github.com/mohuangx/SAVER, and ran with the default settings on the raw count matrix.

##### scImpute

scImpute [18] is an imputation method for scRNA that learns each gene’s dropout probability based on a mixture model, and imputes the probable dropouts based on the information of the same gene from similar cells. scImpute was downloaded from https://github.com/Vivianstats/scImpute and ran on the raw count matrix. The algorithm requires a user-specified parameter specifying the number of cell subpopulations expected (*Kcluster*). For the *in silico* datasets, we ran scImpute with three different *Kcluster* settings reflecting three different cell subpopulation groupings: Lineage class [4], Cell-Type [8], and Sub-Cell Type (14 for H3K4me1/3 and H3K27me3, 13 for H3K9me3).

#### Data smoothing

##### kNN-smoothing 2

kNN-smoothing 2 [19] is a denoising algorithm developed for scRNA data. The algorithm relies on identifying similar cells based on a kNN graph, and then iteratively smooths the counts across neighboring cells. KNN-smoothing was downloaded from https://github.com/yanailab/knn-smoothing. The only required parameter by the algorithm is the number of neighbors considered for the smoothing. We ran the algorithm with three different *k* settings [3, 7, 15], ranging from minimal smoothing (*k*= 3) to larger smoothing (*k*= 15). Apart from that, the algorithm was run with the default settings. The input count matrix underwent library size normalization prior to imputation with kNN-smoothing.

##### MAGIC

MAGIC [15] (Markov Affinity-based Graph Imputation of Cells) is a denoising algorithm commonly applied to scRNA data. It relies on learning the underlying structure of the data through a KNN graph, and shares information across similar cells via data diffusion. MAGIC was downloaded from https://github.com/KrishnaswamyLab/MAGIC. Imputation through MAGIC was run on the default setting on a dataset after library size normalization.

#### Matrix theory

##### ALRA

ALRA [20] (Adaptively thresholded Low-Rank Approximation) is an imputation algorithm for scRNA data based on low-rank matrix approximation. The algorithm was downloaded from https://github.com/KlugerLab/ALRA and ran with the default settings on a dataset after library size normalization.

##### scOpen

scOpen [10] is an imputation method that uses regularized non-negative matrix factorization to impute scATAC data. The algorithm was downloaded from https://github.com/CostaLab/scopen and ran on the raw count matrix with the default settings.

#### Autoencoders

##### DCA

DCA [21] (Deep Count Autoencoder) is a denoising algorithm for scRNA data based on an autoencoder framework. It combines a deep learning autoencoder with specialized loss functions to learn the underlying true zero-noise data manifold. The algorithm was downloaded from https://github.com/theislab/dca and ran with the default settings on the raw count matrix.

##### SCALE

SCALE [16] (Single-Cell ATAC-seq analysis via Latent feature Extraction) is a method for the analysis of scATAC-seq data based on autoencoders. It combines a variational autoencoder framework with a Gaussian Mixture Model to extract the latent features of the data. These can then be used for various tasks such as clustering as well as denoising and imputation of the data. We downloaded the latest version of the algorithm from https://github.com/jsxlei/SCALE, and ran it on the raw count matrix. We ran the algorithm so that we obtained numerical imputed data instead of binary (–impute). For the *in silico* datasets, there were no minimum requirements in the number of peaks per cell, cell per peak and number of variable features.

##### SCALEX

SCALEX [22] is a method for online integration of heterogeneous single-cell data based on variational autoencoders. The algorithm can be used for both data integration and imputation. The latest version of SCALEX was obtained from https://github.com/jsxlei/SCALEX and ran on the raw count matrix. The algorithm was run both with settings for RNA (termed SCALEX.RNA) and ATAC (termed SCALEX.ATAC) single-cell profiles. For the *in silico* datasets, there were no minimum requirements in the number of features per cell or cells per feature.

#### Dictionary learning

##### Cistopic

cisTopic [23] is an algorithm that uses topic modeling to identify cell states and *cis-*regulatory topics (region-topic distribution) in single-cell epigenomic data. While not designed specifically for data imputation, an approach for such use was described in Li *et al.*, 2021 [10].

The algorithm was downloaded from https://github.com/aertslab/cisTopic and ran it with 5 to 50 topics. The optimal number of topics was chosen based on the minimum perplexity. Then the topic cell–cell and region–topic distribution were multiplied to obtain the predictive distribution. This distribution describes the probability of each region in each cell and can be used as an imputed matrix, as proposed by Li *et al.*, 2021.

#### Runtime errors

Some algorithms encountered technical errors in specific *in silico* datasets (Supplementary Tables S3–S6). SAVER failed at low read depth for all marks and did not converge at high read depth for H3K4me1 and H3K27me3. ALRA failures were primarily due to too sparse data for the singular value decomposition step. SCALEX, in contrast, showed poor performance for particular marks but did not encounter technical errors.

### Evaluation metrics

#### Correlation of epigenomic profiles between single-cell *in silico* datasets and ground truth

For each cell, we calculated the Pearson correlation between its single-cell epigenomic profile and the ground-truth profile of the corresponding cell type. We used Pearson correlation because it captures both overall similarity in signal and residual effects of dropout or noise, making it a stringent measure of signal recovery. For datasets prior to imputation, we used the library-size normalized count matrix; for datasets after imputation, we used the imputed count matrix returned by each method. The correlation score for a given method and scenario was summarized as the median correlation across all cells.

#### Signal specificity through signal in peaks scores

To evaluate the specificity of the signal in datasets before and after imputation, we calculated the SiP (Signal in Peaks) scores. In the case of the *in silico* datasets, peaks were called from the original dataset in the same way as described in the original manuscript [3] via the *hiddenDomains* algorithm. The output peaks were filtered by removing peaks with no other nearby peaks within a 50 kb range, merge peaks within 5kb from each other, and filter peaks <10 kb. Finally, we only kept peaks on chromosomes found in all samples, as the peak caller did not always converge for each chromosome.

Since the count matrices are using 50 kb bins, we calculated the SiP based on bins overlapping peak calls or cell type-specific genes.


(2) \begin{eqnarray*}
SiP = \frac{{\textit{Signa}{{l}_{\textit{overlapping}\ \textit{peaks}}}}}{{\textit{Signa}{{l}_{all}}}}
\end{eqnarray*}

To calculate the SiP score for a given imputation (or no imputation) method in each scenario, we calculate the median SiP score across all cells.

Additionally, while these were not taking into account for the ranking, we calculated the SiP scores over the promoter of cell type-specific genes for the H3K4me3 datasets similarly to Lochs *et al.* (2024) [11]. Briefly, H3K4me3 ChIC-counts per cell type were counted per promoter, defined as 2 kb downstream to 500 bp upstream of each transcription start site (TSS) from the UCSC knownGene mm10 table. We took the 250 genes with the highest log2-enrichment compared to all other celltypes as the set of celltype-specific genes.

For the scCUT&Tag enrichment analyses, we downloaded H3K4me3 ChIP-seq peaks of cortical neurons (CN) from Bonev *et al.* (2021) [25] (accession: GSE96107). Intra-dataset cell type-specific peaks were called using the *FindAllMarkers*-function of Seurat 5 [24]. The top-500 peaks per cell type with and FDR < 0.1 were kept for downstream analyses (see Signal Enrichment).

#### Calculating cell–cell similarity

To evaluate clustering effectiveness in single-cell genomics, we developed SIMIC (Similarity-Integrated Metric for Improved Clustering), a method that integrates known or inferred cell-type similarities. This approach is performed directly on the count matrix, bypassing dimensionality reduction techniques such as PCA or UMAP and limiting parameter tuning, ensuring an impartial and data-agnostic assessment.

First, we generated pseudobulks for each cell type by summing counts across bins for all cells within a given type. We then computed cosine distances between these pseudobulks, yielding a distance matrix. The cosine distances are converted into similarity scores, normalized to the range. To account for cell-type abundances, we weighted the similarity matrix using the proportion of cells from each type. The expected similarity between cell types is given by:


(3) \begin{eqnarray*}
E{{S}_{ij}} = \mathop \sum \limits_{i,j} {{W}_i}{{W}_j}{{S}_{ij}}
\end{eqnarray*}

where ${{W}_i}$ and ${{W}_j}$ are the proportions of cell types $i$ and $j$, and ${{S}_{ij}}$ is the similarity score. This provides a baseline

measure of similarity.

To assess clustering quality, we identified the top 5% of highly variable bins and computed a cell-to-cell distance matrix using $( {1 - cor{{r}_{\textit{Pearson}}}} )$. For each cell, we computed the average similarity of its cell type to its 10 nearest neighbors, then calculated the dataset-wide similarity score (${{S}_{obs}}$) by averaging these per-cell scores. Finally, we normalized the dataset-wide score by dividing ${{S}_{obs}}$ by the expected similarity $E{{S}_{ij}}$ to obtain the SIMIC score:


(4) \begin{eqnarray*}
\textit{SIMIC} = \frac{{{{S}_{obs}}}}{{E{{S}_{ij}}}}\
\end{eqnarray*}

This ratio quantifies how well the method improves clustering by comparing observed and expected cell-type similarities. A SIMIC score of 1 indicates that cells are, on average, no more similar to their nearest neighbors in feature space than expected under a random distribution of neighbors. Values greater than 1 reflect improved clustering performance (e.g. a score of 1.5 means neighbors are 50% more similar than expected), whereas scores below 1 indicate worse-than-random clustering.

#### Cluster-prediction performance

To assess whether the imputed datasets enable better clustering, we used a similar KNN-based method as for SIMIC to derive confusion matrices and receiver-operating characteristic (ROC) curves. Instead of calculating the mean similarity score between a cell and its *N* nearest neighbors, we determined the enrichment of clusters compared to the known total proportion in the dataset. The most abundant cell type was assigned as the predicted cell type for that particular cell. Using this predicted cell type, we compared it to the true cell type of each cell, constructing confusion matrices and generating ROC plots to evaluate the predictive performance of the clustering method.

#### Calculating algorithm scores and ranking

To rank the imputation algorithms, we used a similar ranking method to that used in Luecken *et al.*, 2022 [26]. To ensure that each metric is equally weighted and has the same dynamic range, we min-max scaled the median output of every metric (*Y*) per algorithm across the same scenario:


(5) \begin{eqnarray*}
\textit{scaled}\left( Y \right) = \frac{{Y - \min \left( Y \right)}}{{\max \left( Y \right) - \min \left( Y \right)}}
\end{eqnarray*}

Those algorithms that failed to run in a specific scenario (either due to technical failures, excessive memory requirements (over 30 Gb) or running time (over 48 h) were given a scaled score of 0 across all metrics for that specific scenario.

Scaled metrics were grouped in three tasks in order to calculate task-specific scores: (i) similarity to ground truth dataset (*S*_correlation_), evaluated though the correlation to ground truth; [2] similarity across cells from the same subpopulation in comparison to those of other subpopulation (*S*_similarity_), evaluated through the similarity scores calculated on the count matrix; and [3] specificity of signal enrichment (*S*_enrichment_), evaluated through the called peaks FRiP scores.


(6) \begin{eqnarray*}
{{S}_{\textit{correlation}}} = \textit{scaled}\left( {\textit{correlation}} \right)
\end{eqnarray*}

(7) \begin{eqnarray*}
{{S}_{\textit{similarity}}} = \textit{scaled}\left( {\textit{similarity}} \right)
\end{eqnarray*}

(8) \begin{eqnarray*}
{{S}_{\textit{enrichment}}} = \textit{scaled}\left( {Si{{P}_{\textit{peaks}}}} \right)
\end{eqnarray*}

A scenario-specific score (*S*_scenario_) was then calculated for each algorithm based on the mean of these task-specific scores:


(9) \begin{eqnarray*}
{{S}_{\textit{scenario}}} = \frac{{{{S}_{\textit{correlation}}} + {{S}_{\textit{similarity}}} + {{S}_{\textit{enrichment}}}}}{3}
\end{eqnarray*}

Finally, an overall score (*S*_overall_) was calculated based on the median *S_scenario_* across all scenarios:


(10) \begin{eqnarray*}
{{S}_{\textit{overall}}} = med\left( {{{S}_{\textit{scenario}}}} \right)
\end{eqnarray*}

Algorithms were ranked both in a scenario-specific and overall manner based on the *S*_scenario_ and *S*_overall_, respectively.

### Signal enrichment

Enrichment computations were performed on the raw input matrices using the tidyCoverage package [42]. Peaks with <50 kb separation were merged together. 1000 peaks were randomly selected per mark, and the signal coverage was calculated within a range of ± 0.5Mb around the center of each peak (for H3K4me3 and H3K4me1) or peak start coordinates (for H3K9me3 and H3K27me3). Within each of these genomic regions, we normalized the signal by the average coverage within each genomic region.

### Dimensionality reduction

Before performing Uniform Manifold Approximation and Projection (UMAP), we applied principal component analysis (PCA) for dimensionality reduction. The input for PCA included both read depth-normalized (counts per million) datasets and the raw output of the imputation algorithms. We iteratively determined the latent dimension that best separated clusters, using the silhouette score as a loss function to optimize several parameters for both PCA and UMAP.

We varied the percentage of most variable bins (90% or 95%) used for PCA, the number of principal components (PCs) considered $[ {5,\ 50} ]$, and the local neighborhood size for manifold approximation $[ {10,15,20} ]$. The parameters yielding the highest average silhouette score in UMAP space were selected for the final UMAP calculation (see Supplementary Table S7 for final parameters).

## Results

**Figure 2.**
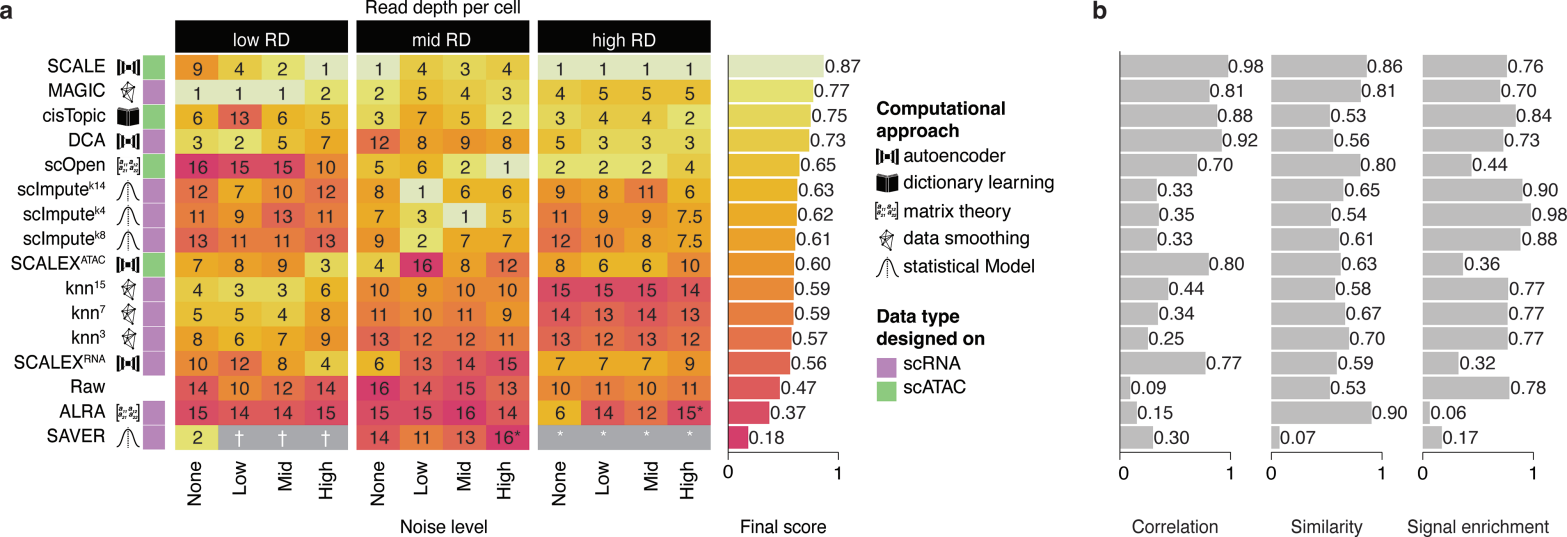
*In silico* benchmarking on simulations from sortChIC H3K4me3 data. (**A**) Ranking of the imputation algorithms across several *in silico* datasets generated from bone marrow sortChIC for H3K4me3. Several combinations of read depth per cell and noise were tested. Algorithms are ordered from top to bottom according to their median score across all scenarios. On the left side of the graph, each algorithm is classified according to the type of computational approach behind it, and the data type they were designed on (scRNA or scATAC). On the right side, we can see both their final score across all scenarios. (**B**) Score per task for each algorithm. Algorithms are ordered in the same manner as in (A).

**Figure 3.**
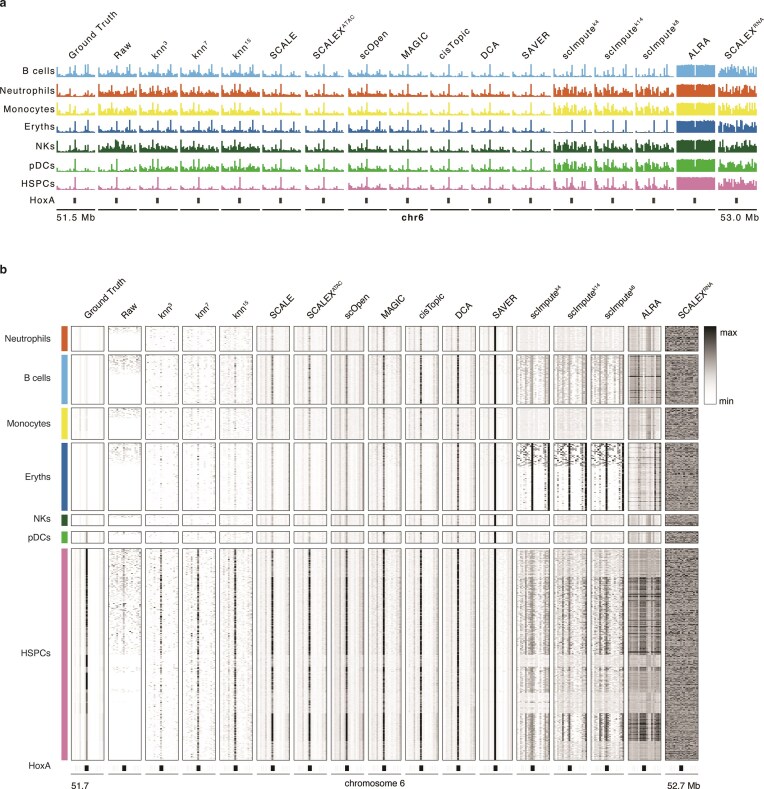
Imputation algorithms produce variable output on *in silico* sortChIC H3K4me3 data. (**A**) Pseudobulk tracks H3K4me3 signal in datasets with 1000 reads/cell and 1× noise over the Hoxa gene cluster for different cell types and imputation algorithms. (**B**) Single-cell matrices of the same datasets as in (A) over the Hoxa gene cluster.

**Figure 4.**
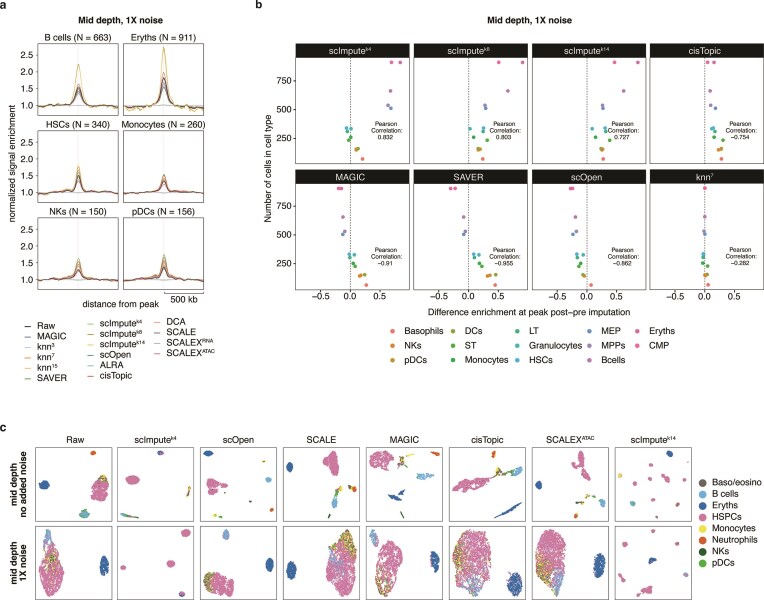
Task-specific effects of imputation algorithms on *in silico* sortChIC H3K4me3 data. (**A**) Signal enrichment plot over 1000 randomly selected H3K4me3 peaks ± 0.5 Mb in datasets with 1000 reads/cell and 1× noise. Signal enrichment over relevant peaks was calculated for six cell subpopulations: B cells, Eryths, HSCs, Monocytes, NKs, and pDCs. *N* indicates the number of cells per subpopulation. Different colors indicate the imputation algorithm used. (**B**) Difference in signal enrichment pre- and post-imputation at those peaks against number of cells per cell type. Different colors indicate the specific cell type. (**C**) UMAP representation of datasets with 1000 reads/cell and 1× noise. A subset of datasets were selected: the dataset pre-imputation and 7 datasets after imputation with the top 7 performing algorithms for this scenario.

**Figure 5.**
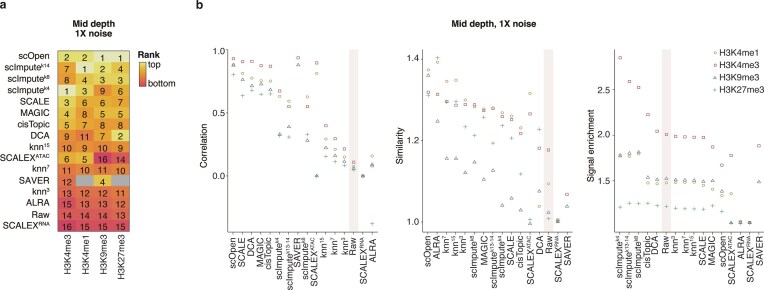
Performance comparison *In silico* comparison of imputation algorithms across different epigenetic marks. (**A**) Ranking of the imputation algorithms across *in silico* datasets generated from bone marrow sortChIC datasets for H3K4me3, H3K4me1, H3K9me3, and H3K27me3. Three scenarios are shown with mid read depth per cell and 1× noise.The algorithms are ordered from top to bottom performers according to their median rank across epigenetic marks. (**B**) Task-specific scores per algorithm per mark for mid read-depth per cell and 1× noise.

**Figure 6.**
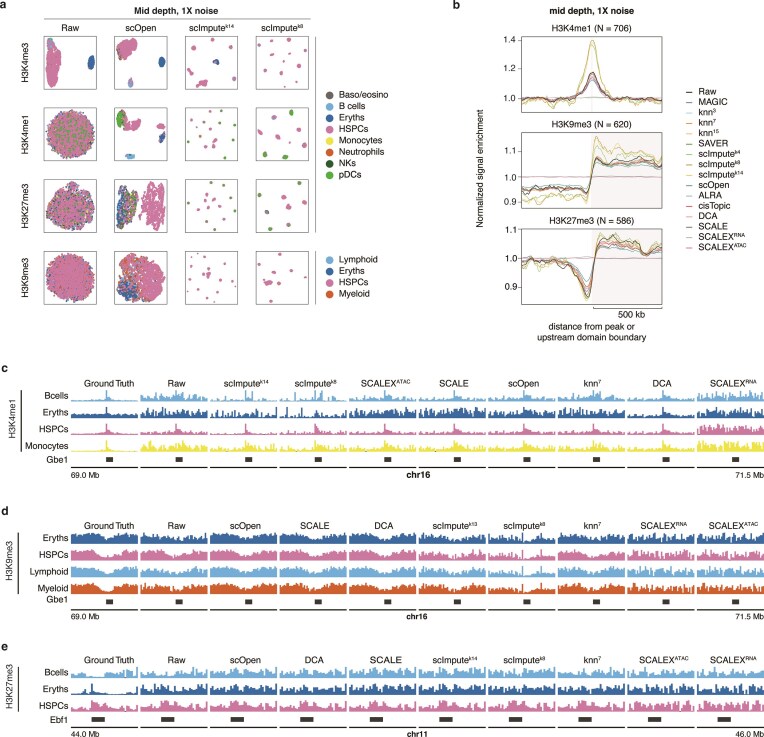
Task-specific effects of single-cell imputation algorithms across different epigenetic marks. (**A**) UMAP representation of H3K4me3, H3K4me1, H3K27me3, and H3K9me3 datasets with 1000 reads/cell and 1× noise. A subset of datasets were selected: the dataset pre-imputation and three datasets after imputation with the top 2 performing algorithms for this scenario (scOpen, scImputek14, and scImputek8). (**B**) Signal enrichment plot over 1000 randomly selected H3K4me1, H3K9me3, and H3K27me3 peaks in B cells for datasets with 1000 reads/cell and 1× noise. For H3K4me1 peaks, we show the signal enrichment at the peak center ± 0.5 Mb. For H3K9me3 and H3K27me3, we show the signal enrichment ± 0.5 from the domain boundary start coordinate. Different colors indicate the imputation algorithm used. Pseudobulk signal in datasets with 1000 reads/cell and 1× noise over the Ebf1 locus for (**C**) H3K4me1 and (**D**) H3K9me3, and over the Gbe1 locus for (**E**) H3K27me3.

**Figure 7.**
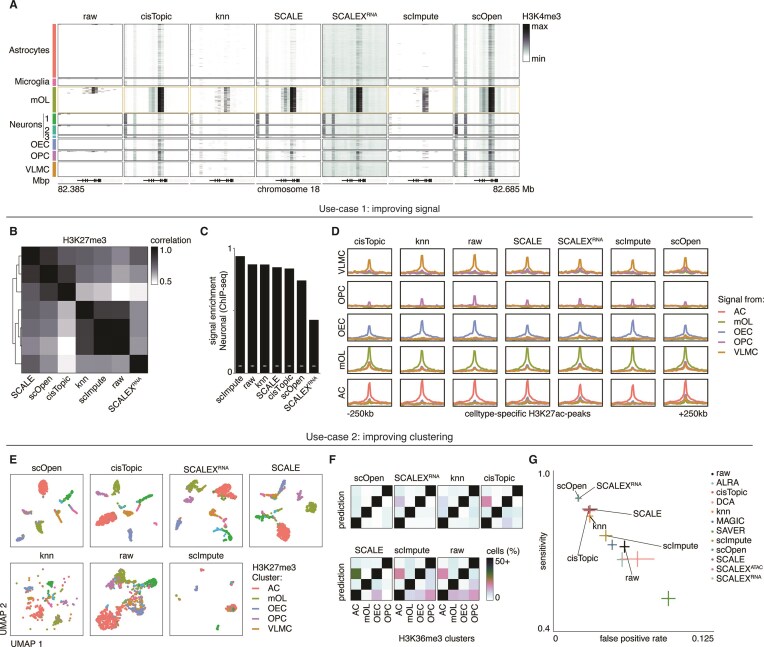
Imputation of scCUT&Tag shows dichotomy in use-cases. (**A**) Example single-cell heatmap of the Mbp-locus. Cells are divided across nine subpopulations: Astrocytes; Microglia; mature oligodendrocytes (mOLs); Neurons 1, 2, and 3; olfactory ensheating cells (OECs), oligodendrocyte progenitors cells (OPCs), and vascular and leptomeningeal cells (VLMCs). Mbp is expressed specifically in the mOL-cluster (gold borders). Signal is minmaxed per method. (**B**) Spearman correlation of pan-algorithm H3K27me3 pseudobulk profiles (concatenated bin × cluster RPKM values). (**C**) Fraction of Signal In Peaks of H3K4me3 signal from cells in the Neuron-1, -2, and -3 clusters on ChIP-seq peaks of cortical neurons. White bars indicate the enrichment of randomized samples (25th and 75th quantile of a 1000 permuted peak sets). (**D**) Signal enrichment profiles of scCUT&Tag pseudobulk-signal per cluster on cell type-specific peaks. (**E**) Optimized UMAP representation of all methods on H3K27me3. Colors denote original cell types. (**F**) Confusion-matrices of H3K36me3 based on *K* = 10 nearest neighbors prediction. (**G**) ROC plot of the average sensitivity and FPR per method across the modalities.

To assess the effectiveness of these imputation methods on scHPTM data, we focused on the two main cases in which imputation may be beneficial: improving signal quality and enhancing cell–cell clustering accuracy (Fig. 1C). For our evaluation, we used *in silico* scHPTM datasets from mouse bone marrow generated with sortChIC [3] (Figs 1–6 and Supplementary Table S1) and froam mouse brain generated via scCUT&Tag [4] (Fig. 7 and Supplementary Table S2). Both techniques rely on antibody binding to specifically profile HPTMs, followed by enzymatic cleaving (through MNase in sortChIC, and the Tn5 transposase in scCUT&Tag). Both datasets capture scHPTM data for active promoters (H3K4me3 and/or H3K27ac), enhancers (H3K4me1 and/or H3K27ac), and repressed loci (H3K27me3 and/or H3K9me3). Additionally, the scCUT&Tag dataset includes H3K36me3 data, which marks actively transcribed gene bodies.

### Performance-metrics and ranking

First, we assessed how well the imputed single-cell profiles matched the true underlying biological epigenetic signatures (Fig. 1C). By calculating the Pearson correlation between each cell’s imputed profile and the corresponding ground truth sample, we could quantify the linear relationship and similarity to the known biological signal.

In addition to similarity to ground truth, we also evaluated the imputation methods’ ability to improve similarity between cells (i.e. clustering) (Fig. 1C). Other imputation benchmarking studies rely on clustering metrics such as the Adjusted Rand Index (ARI) or silhouette scores, which require the user to perform dimensionality reduction and/or clustering [14, 27]. To avoid biases caused by additional parameter choices, we developed the custom metric SIMIC (Similarity-Integrated Metric for Improved Clustering) (Materials and methods). This metric captures the similarity between nearby cells and compares it to an *a priori* generated cell type similarity-matrix. SIMIC was calculated before dimensionality reduction to minimize the effects of nonoptimal downstream analyses (e.g. number of PCs selected, filtering of noninformative signal). This approach is similar to the recently proposed scGraph [28], which also takes advantage of the similarities between cell types to evaluate cell embeddings.

Finally, we quantified the signal enrichment (Fig. 1C)—the proportion of the total imputed signal that fell within the ground truth peaks, as defined in the original manuscript [3]. This Signal in Peaks’ (SiP) score provides insight into how well the imputed signal was able to recapitulate the genuine regulatory regions and epigenetic activity.

These three key metrics were used to gain a comprehensive understanding of how each imputation algorithm performed across the critical dimensions that impact downstream data quality and biological interpretation. We included these metrics in the accompanying R-package for this paper (Fig. 1A and C). The scores for these metrics were scaled, averaged, and used to rank [26] the relative effectiveness of the different imputation approaches, both within each task and overall.

### Controlling noise and sparsity with *in silico* datasets

Data quality in scHPTM profiling can vary widely across protocols, epitopes, and even between individual experiments, making it essential to control for these factors during benchmarking to ensure accurate interpretations. To address this, we generated *in silico* scHPTM datasets based on the sortChIC datasets, with varying read depths (100, 1000, and 10 000 reads per cell) and noise levels (Fig. 1A and Supplementary Fig. S1; Materials and methods). Noise is defined as the level of added random signal according to the genome-wide coverage in the ground truth profiles (Materials and methods).

Our analysis confirmed that datasets with different read depths retain signal enrichment at similar loci (Supplementary Fig. S1C), despite exhibiting clear differences in sparsity (Supplementary Fig. S1A). Moreover, we confirmed that signal enrichment declines noticeably in datasets with higher noise levels (Supplementary Fig. S1B and C), as expected. Both depth and noise influence the signal specificity, as shown by the percentage of genomic bins with signal across the genome (Supplementary Fig. S1). These results confirm that our *in silico* datasets provide a robust foundation for evaluating imputation methods across various data quality scenarios.

### Overall performance on H3K4me3 *in silico datasets*

We first focused on evaluating the performance of the selected algorithms using the generated *in silico* H3K4me3 datasets. H3K4me3 marks the transcription start sites of active genes and has been shown to preferentially label genes essential for cell identity and cell type-specific function [3, 29]. This made H3K4me3 particularly suitable for the initial benchmarking as, even with added noise and lower read depth, the *in silico H3K4me3* datasets would still contain cell type-specific information that could be used by the imputation algorithms.

All imputation methods were performed on the *in silico* H3K4me3 datasets. However, not all algorithms completed successfully across all scenarios. Both ALRA and SAVER failed in certain cases (Fig. 2A; Supplementary Figs S2 and S3, and Supplementary Table S3). ALRA failures were primarily due to too sparse data for the singular value decomposition step. SAVER failed in scenarios with low read depth, and in some mid-read-depth scenarios with high noise. At high read depth, SAVER completed successfully in ~25% of runs, while the remaining runs reached time or memory limits. By evaluating the resulting imputed datasets from the different scenarios, we were able to assess the relative strengths and weaknesses of each imputation approach and rank their overall performance.

Most algorithms improved overall performance (i.e. the combined rankings of individual task-scores) compared to the nonimputed datasets (Fig. 2A), regardless of read depth and noise levels. Depth emerged as the primary determinant of performance across all metrics, with noise levels having a secondary impact (Fig. 2A, and Supplementary Figs S2 and S3). Notably, SCALE, MAGIC, and cisTopic consistently ranked among the top performers across several scenarios (ranked first, second, and third overall, resp.). No single computational principle significantly outperformed the others on overall rankings (Kruskal–Wallis test, *P*= 0.72) (Supplementary Fig. S4A). Interestingly, methods developed for scATAC data showed higher effectiveness (Wilcoxon signed rank test, *P*< 0.05) (Supplementary Fig. S4B), suggesting that scATAC-based algorithms could potentially be applied to scHPTM datasets with narrow peaks, such as H3K4me3.

### Task-specific performance on H3K4me3

To better understand the strengths and limitations of each method, we examined their scores on the *in silico* H3K4me3 datasets across the three key evaluation tasks: biological profile similarity (Pearson correlation), cell–cell similarity (SIMIC score), and signal enrichment (SiP score). Biological profile similarity was assessed by calculating the Pearson correlation with ground-truth profiles, showing that correlation generally improved after imputation (Fig. 2B, and Supplementary Fig. S2A and B). However, scOpen and ALRA had lower scores in high-depth, low-noise scenarios, suggesting that while imputation can improve similarity to the true signal, it may introduce inaccuracies when data quality is already high.

When quantifying improvements in cell–cell similarity (i.e. enhancing the input for clustering algorithms) using SIMIC, substantial gains were only observed in mid-read depth scenarios for most methods (Supplementary Fig. S2C and D). This suggests that imputation can improve cell–cell similarity when there is enough data (i.e. mid-depth datasets), but it has an upper limit. As noise levels increase or read depth decreases, performance drops substantially, indicating that imputation methods struggle to improve clustering in these scenarios. These results highlight that imputation is most effective for this task when there is sufficient information to work with, but it cannot compensate for poor data quality or low sequencing depth.

For the third task, improving signal enrichment at known loci, we compared the SiP-scores over ground-truth peaks for each cell type, derived from the original dataset [3] (Supplementary Fig. S3A and B). As expected, SiP-scores in the nonimputed (i.e. input) datasets were not influenced by depth but decreased as noise levels increased. After imputation, performance varied across algorithms. At high and mid read depth, scImpute and cisTopic improved SiP-scores, with the most significant improvement seen at mid read depth (∼0.80, ∼0.67, and 0.67 for scImpute.k14, cisTopic, and no imputation respectively, at mid read depth, 0× noise). scOpen underperformed in high read depth scenarios (Supplementary Fig. S3A and B), something we also found in the correlation-based analyses. We also observed similar trends when calculating SiP scores over cell type-specific promoters (Supplementary Fig. S3C and D). This confirmed that imputation with scImpute or cisTopic can enhance signal enrichment in mid read depth H3K4me3 datasets.

Taken together, while some algorithms consistently outperform the rest, their performance across different combinations of read depth per cell and noise can be variable (Fig. 2A). This variability suggests that the best algorithm for a given dataset depends on its specific characteristics. For example, some algorithms, such as SCALEX, show unstable performance across scenarios, occasionally obtaining bottom-tier performance (Supplementary Figs S3 and 4).

To visualize the effects of imputation algorithms, a specific scenario of mid read depth and -noise was selected. Pseudobulk tracks for the *Hoxa* gene cluster (Fig. 3A), a region also explored in the original paper [3], were examined. The H3K4me3 signal was expected to be mainly present in progenitor cell types (HSCs), and absent in more differentiated cell types. Most algorithms enriched the signal across all cell types, though scImpute and KNN maintained low signal in differentiated cell types, highlighting their accuracy. Further examination of the single-cell matrices (Fig. 3B) confirmed that the signal is spread across most cells within a given cell type after imputation, with intra-cluster variability reduced for most algorithms.

Signal enrichment ± 0.5 Mb around the ground-truth peak calls for B cells, erythrocytes (Eryths), hematopoietic stem cells (HSCs), monocytes, natural killer (NK) cells, and plasmacytoid dendritic cells (pDCs) were also visualized (Fig. 4A). This supported our previous findings: most methods reduced SiP-scores for larger clusters but improved SiP for smaller ones. To follow up on this observation, we plotted the difference in signal enrichment at ground truth peaks per cell type before and after imputation against the number of cells (Fig. 4B and Supplementary Fig. S5A). This showed that, for most algorithms, there is a strong correlation (positive or negative) between the two variables. To determine whether this is solely dependent on the number of cells per cell type, or if it is influenced by dataset imbalance, we simulated a dataset from the same scenario in which all cell types were of equal size (89 cells) (Supplementary Fig. S5B). This analysis showed that, while some variation in signal enrichment after imputation is inherent to the nature of each cell type, it becomes accentuated according to cell type size within an unbalanced dataset for certain algorithms (SAVER, scImpute, cisTopic, and SCALEX).

Finally, the nonimputed dataset and outputs from the top seven methods were visualized using UMAP at both no- and mid-noise levels (Fig. 4C). While scOpen and SCALE improved cell type separation, scImpute variants increased the mixing of subpopulations.

In summary, some algorithms excel at specific tasks—namely scOpen and SCALE for enhancing clustering and scImpute for signal enrichment—while none perform well across all tasks. This indicates that the optimal imputation algorithm for a given dataset will depend on its specific characteristics and the intended downstream analysis tasks. Comparing performance across different epigenetic marks.

### Performance across epigenetic marks

Epigenetic modifications vary in many different features such as domain size, genomic distribution, and accessibility. Therefore, we next sought to investigate the effects of these differences on imputation performance. We generated *in silico* H3K4me1, H3K27me3, and H3K9me3 datasets for all scenarios. All imputation methods were run on these datasets and scored on the same tasks as before (Supplementary Figs S6 and  7). Some algorithms encountered technical errors or performed poorly for specific histone marks (Materials and methods).

While general rankings remained relatively stable across scenarios (Fig. 5A, and Supplementary Fig. S8A and C), scATAC-based methods outperformed scRNA-based methods specifically on H3K4me1 datasets (Wilcoxon signed rank test, *P*< 0.05) (Supplementary Fig. S9B). In contrast, no single computational principle significantly outperformed the others on overall rankings across all the other epigenetic marks (Kruskal–Wallis test, *P*= 0.72, 0.92, and 0.44 for H3K4me1, H3K9me3, and H3K27me3, respectively) (Supplementary Fig. S9A). For H3K27me3, which features both narrow peaks and broad domains, the difference was marginal (*P*< 0.1), while no significant effect was observed for the broadly deposited H3K9me3. This aligns with our earlier prediction that HPTM datasets with predominantly narrow peaks may benefit more from scATAC-based methods.

We therefore wondered what the task-specific differences are between HPTMs. All marks showed improved signal correlation to ground truth, with H3K4me1 and H3K4me3 benefiting the most (Fig. 5B and Supplementary Fig. S8B). For cell–cell similarity and clustering, scOpen consistently outperformed other methods across all HPTMs as evidenced by the SIMIC scores, being only outperformed by ALRA for H3K4me1 and H3K27me3 (Figs 5B and 6A, and Supplementary Fig. S10). Interestingly, H3K9me3 imputation underperformed on cell–cell similarity in all other algorithms, highlighting the inherent challenges in clustering data from this mark [3, 11]. Both H3K4me1 and H3K9me3 outputs from scImpute showed increased signal enrichment within known peaks (SiP score) compared to the datasets pre-imputation (Figs 5B and 6B, and Supplementary Fig. S8B). This effect was not seen for H3K27me3, suggesting that enriching true signal in datasets containing domains of diverse widths (Supplementary Fig. S8B) remains a challenge for current methods. These results show that while rankings between HPTMs are broadly similar, the extent of improvement is specific to each HPTM.

To exemplify these results, we focused on known cell type-specific regions. Previous work has shown that the *Gbe1* locus (a key transcription factor for B cell cell-fate specification) is specifically depleted for H3K4me1 in Erythroblasts, which is instead more tightly surrounded by H3K9me3 [3]. This made this region a prime candidate to inspect the performance of both HPTMs. While most algorithms faithfully imputed the H3K4me1 signal in B cells (Fig. 6C and Supplementary Fig. S11A), the H3K9me3 signal lost most cell type-specific signal (i.e. the width of the depleted locus) (Fig. 6D and Supplementary Fig. S11B). Similarly, at the *Ebf1* locus [30], which has strong H3K27me3 deposition in HSPCs, all methods except SCALEX successfully reconstructed the cell type-specific signal (Fig. 6E and Supplementary Fig. S11C). These loci reinforce our earlier results on intra-cluster similarity (Fig. 5B): the success of cell type-specific signal reconstruction varies by HPTM, and caution should be exercised before presenting imputed data as conclusive.

### Imputation on scCUT&Tag data

We next sought to verify our observations in a secondary dataset. For this, we used the scCUT&Tag dataset from Bartosovic *et al.* (2021) [4], which is a similar approach to sortChIC and produces data which also contains high signal-to-noise ratios. This dataset contains single-cell data from the mouse brain at 10 kb resolution for four HPTMs: H3K27ac, H3K27me3, H3K36me3, and H3K4me3. Based on our results from the *in silico* analyses, we focused on the top-performing methods for computational efficiency.

Visual inspection at cell type-specific marker loci shows consistent outcomes across methods when compared to the *in silico* results. H3K4me3 signal at the *Mbp* locus, which is expressed in mature oligodendrocytes (mOLs), is faithfully imputed by most methods, though with different levels of variation (Fig. 7A). KNN and scImpute add signal specifically to genomics bins overlapping with *Mbp* in the mOL cluster but introduce a level of cell-to-cell variability that is not verifiable in the original dataset. Conversely, cisTopic, SCALE, SCALEX, and scOpen appear to artificially remove cell-to-cell variation and fill data in these bins. For this second set of methods, signal is also present at the *Mbp* locus in other clusters, resulting in the loss of cell type specificity.

Given that imputation methods vary in their ability to perform two distinct tasks—signal enrichment and separation of cell subpopulations—we tailored our downstream analyses to these tasks. First, we aimed to quantify which methods retain a signal most similar to the input data while imputing missing data in a cell type-specific manner. To assess whether the imputed data remains comparable at the coarse-grained cell type level, we performed correlation analysis of combined pseudobulk tracks for each HPTM. For H3K27me3, KNN and scImpute showed high correlation with the raw data (0.86 and 0.88, respectively), while SCALE, scOpen, and cisTopic exhibited lower correlations (0.77, 0.76, and 0.68, resp.) (Fig. 7B). Moreover, scImpute was the only method that improved H3K4me3 signal enrichment for neuronal cells over public ChIP-seq peaks [25] (Fig. 7C). We also called cluster-specific peaks and computed signal enrichment for each peak set, allowing us to quantify differences in signal between methods and assess signal specificity (Fig. 7D). We further assessed signal specificity by comparing signal enrichment within the relevant sets of cluster-specific peaks (intra-cluster signal) to signal enrichment within sets of peaks from other clusters (inter-cluster signal). While cisTopic, scImpute, and KNN showed comparable or higher intra-cluster signal, only scImpute reduced inter-cluster signal levels (Supplementary Fig. S12A). These findings indicate that scImpute was the only method that improved the signal of the original dataset.

The second task focused on improving clustering by reducing the distance between cells of the same or similar cell types while increasing the distance between cells of dissimilar cell types. This improvement brings clusters closer to the ground truth, making downstream analyses more robust. Inspection of the UMAPs revealed that SCALE, SCALEX, scOpen, and cisTopic improved cell type clustering (Fig. 7E and Supplementary Fig. S12B). This was supported by the relative log2 increase in SIMIC scores across HPTMs: SCALE (0.40 ± 0.19), SCALEX (0.44 ± 0.16), scOpen (0.44 ± 0.16), and cisTopic (0.40 ± 0.17). KNN and scImpute only showed slight improvements by the differential SIMIC score (0.38 ± 0.13 and 0.26 ± 0.18, respectively) (Supplementary Fig. S12C), illustrating the limited utility of latent dimensions for imputation. We then evaluated clustering accuracy by predicting cell types using KNN-based overrepresentation. scOpen- and SCALEX-derived matrices produced good predictions across HPTMs (Fig. 7F and G, and Supplementary Fig. S12D). However, the performance of SCALE-, KNN-, and cisTopic-based matrices was more variable, primarily due to reduced sensitivity and specificity between closely related cell types (Fig. 7F and G, and Supplementary Fig. S12D) and between cell types with overlapping latent dimensions in the original manuscript. In summary, none of the applied methods were able to improve both signal and clustering in scCUT&Tag data. However, scOpen and SCALEX can be used to enhance clustering, after which the raw data can be utilized for downstream analyses.

In this study, we have evaluated the performance of selected imputation algorithms on scHPTM data using *in silico* and publicly available datasets. While none of the currently existing imputation methods outperform all others across all tasks, some consistently excel in a task-specific manner (e.g. scImpute for signal enrichment and scOpen for improving cell–cell similarity).

## Discussion and conclusion

Here, we evaluated how well different imputation algorithms perform on scHPTM data. We covered a range of computational approaches, including statistical modeling, matrix factorization, data smoothing, autoencoders, and topic modeling. We found that imputation performance is influenced by scHPTM-specific factors, including per-cell read depth, noise levels, and the specific epigenetic mark. While no single approach was consistently the best, several of the top-performing methods for narrow-peak HPTMs were originally developed for scATAC-seq data. This suggests that tailoring methods for specific HPTMs may give better results, particularly when differentiating between HPTMs distributed in narrow- and wide domains.

**Figure 8.**
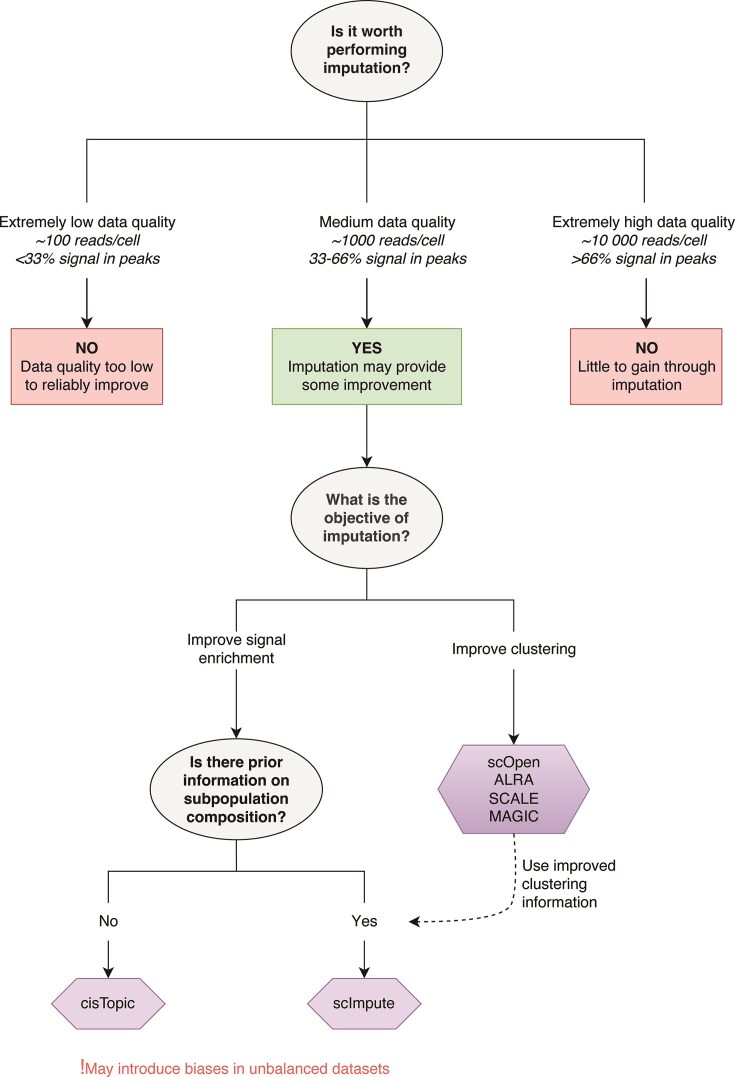
Decision tree for the selection of single-cell imputation algorithms.

Variation in performance makes it hard to recommend any one method as the best for scHPTM data. Instead, some algorithms performed well in specific tasks: for example, scImpute variants improved signal enrichment, particularly for larger cell types, while scOpen was better at separating cell types. Despite the lack of one all-encompassing method for low-quality data, task specificity allows for application in certain cases. If cell types or treatments are known in advance (for instance in screens or multimodal setups), users may focus on imputation to improve signal enrichment. Alternatively, optimizing clustering first and then creating pseudobulks from nonimputed data may help with downstream analyses (Fig. 8).

Our *in silico* dataset generation strategy was designed to isolate and control the effects of both sequencing coverage and background noise on scHPTM data for imputation benchmarking. The use of *in silico* datasets not only allowed us to control the read depth and noise levels per cell but also provided us with a ground truth to compare the imputation output. While coverage effects have been studied in scRNA-seq benchmarking [14], the influence of background noise on scHPTM data has received less attention. Noise in scHPTM data can arise from technical limitations such as nonspecific antibody binding, inefficient DNA fragmentation, or degradation. A substantial portion of this technical noise reflects an open chromatin bias, which is often cell type-specific and difficult to distinguish from true signal. This type of noise might be better addressed by normalizing based on cell type-specific open chromatin profiles. To approximate a more general noise effect, however, we added random uniform noise genome-wide, modeling the type of noncell-specific noise that may be more amenable to computational denoising techniques. Although more complex noise models could be applied, we found that this simple strategy already provides useful insights into how noise impacts the performance of different algorithms

Beyond coverage, noise, and bin size, the structure of scHPTM data itself also shapes the transferability of methods originally developed for scRNA-seq or scATAC-seq. A key distinction is that RNA and ATAC data are typically aggregated over well-defined genomic features (exons or peaks), producing sparse matrices with discrete and relatively independent entries. For scHPTM, such predefined domains are less robust, since peak- or domain-calling is highly dependent on the target and data quality. In practice, what is most informative for scHPTM is often the distribution of signal across the linear genome itself, rather than only enrichment over predefined peaks. This necessitates imputing signal across genomic bins. This distinction has practical consequences: when the signal derives from punctuated, narrow-peak HPTMs and the bin size approximates peak width, the resulting matrix is highly sparse and structurally similar to an ATAC peak-by-cell matrix, which likely explains why scATAC-derived methods performed best in these cases. Conversely, broad-domain HPTMs yield dense matrices in which neighboring bins fragment large domains into many correlated features, a structure that is poorly matched to the assumptions of most ATAC- or RNA-based methods.

Previous benchmarking papers focus on benchmarking scRNA [14, 27, 31, 32] or scATAC [33] data and rely mainly on clustering-related metrics similar to ARI or silhouette scores. Since scHPTM data are not used only for clustering, but also for peak calling and other specialised downstream tasks, we evaluated imputation performance using a combination of metrics evaluating three aspects: cell-cell similarity, signal enrichment, and similarity to a computationally generated ground truth. The use of diverse metrics for scoring means that an algorithm cannot score highly by simply optimizing for a single task. Instead, consistently high scores across all metrics are prioritized, with low performance within a specific aspect being penalized. It should be noted that some of the imputation algorithms can also be used for dimensionality reduction, clustering and further analysis. However, using algorithm-specific methods for dimensionality reduction might result in the scores reflecting the efficiency of dimensionality reduction and clustering approaches instead of measuring the performance of data imputation itself. To avoid this, we calculated all scores directly on raw or read-depth normalized matrices. Furthermore, no feature selection was performed prior to imputation or evaluation, as other studies have shown that feature selection for scHPTM data is detrimental for further analysis [34]. Altogether, this study presents novel metrics for evaluating the performance of imputation on epigenomic data without the need for dimensionality reduction or feature selection.

Our methods for creating *in silico* datasets and evaluating scHPTM imputation performance are made available through SCIBED, an R package for easy use. This work will benefit both method developers who want to compare their new tools with existing ones, and researchers looking to find the best approach for their datasets.

While some existing algorithms show good performance on scHPTM data for specific tasks, there are still many opportunities for further improvement. For example, no current algorithm incorporates information about neighboring genomic bins, which could be very useful for scHPTM imputation. This has been achieved through the use of hypergraphs to impute contact maps from scHi-C data [35], where cells and nonzero bins are considered separate nodes. Contacts between two genomic loci in a cell are represented through hyperedges. The resulting hypergraph is then used to train a neural network for imputation. In scHPTM data, a similar logic could be used to build a hypergraph based on connections of neighboring zero/nonzero bins. The number of genomic bin nodes per hyperedge would depend on the nature of the histone mark. However, it could be determined either from prior knowledge or through training at the pseudobulk level. Furthermore, many scHPTM datasets are accompanied by other readouts that provide information on cell type specification (either an RNA [1, 36, 37]/protein [38] readout or FACS(3) information). ScImpute is the only algorithm that uses prior information on the number of expected cell subpopulations and performs particularly well at signal enrichment, so it might be worthwhile developing other algorithms that also take advantage of this type of information. Alternatively, direct integration of multi-modal information could be used for cross-modality data imputation. This approach has already been applied in several algorithms for imputing data between DNA accessibility and RNA readouts [39, 40, 41]. To our knowledge, no methods have been developed for scHPTM data. However, due to the interconnected nature of the epigenomic code, leveraging multi-modal data across histone modifications could be particularly promising.

## Software

The SCIBED R package is available on https://github.com/KindLab/SCIBED and https://doi.org/10.5281/zenodo.17570174.

## Supplementary Material

lqaf192_Supplemental_Files

## Data Availability

All the *in silico* datasets generated are available on Zenodo (doi: 10.5281/zenodo.14205078). The public datasets used in these benchmark study are the sortChIC datasets for H3K4me3, H3K4me1, H3K9me3, and H3K27me3 in mouse bone marrow (requested to the authors by personal correspondence); and the scCUT&Tag datasets for H3K4me3, H3K27me3, H3K27ac, and H3K36me3 for mouse brain (raw data available in GEO under GSE163532). The H3K4me3 ChIP-seq peaks of cortical neurons (CN) were available in GEO (GSE96107).
