# Supplementary material for: A computational framework to dissect imputation strategies for single-cell histone modification data
Source: NAR Genom Bioinform. 2025 Dec 29;7(4):lqaf192. doi: 10.1093/nargab/lqaf192 (PMC12746105; doi:10.1093/nargab/lqaf192)
Supplement: lqaf192_Supplemental_Files [file lqaf192_supplemental_files.zip › suppfigs.pdf]

Supplementary Figure S1. Generating *In silico* datasets from H3K4me3 sortChIC data.

(A) Single-cell *in silico* matrix profile for B cells over chromosome 1 at 3 different read depth per cell (high - 10,000 reads/cell, mid - 1,000 reads/cell, and low 100 reads per cell) and no added noise. Bottom track shows the single-cell matrix profile for B cells in the original sortChIC dataset. Below is the location where the relevant peaks map to. (B) Single-cell *in silico* matrix profile for B cells at mid read depth per cell and 12 different levels of noise. The top track shows the single-cell matrix profile for B cells in the original sortChIC dataset. At the bottom is the location where the relevant peaks map to. (C) Signal enrichment plot over 1000 randomly selected H3K4me3 peaks in B cells for *in silico* and the original datasets with different read depth and noise levels. d, Fingerprint plot of the different read depth per cell and noise levels.

Supplementary Figure S2. Correlation to Ground Truth and similarity for the *in silico* datasets from sortChIC H3K4me3 data.

Scores per task for every *in silico* H3K4me3 dataset pre- and post-imputation. *In silico* datasets have 3 read depth per cell levels (high - 10,000 reads/cell, mid - 1,000 reads/cell, and low 100 reads per cell) and 12 noise levels. Noise levels were grouped into none, high, mid, low and extremely low. The median Pearson correlation to the ground truth was shown for (A) all scenarios, and (B) grouped per read depth per cell. The median similarity score was shown for (C) all scenarios, and (D) grouped per read depth per cell.

Supplementary Figure S3. Signal enrichment for the *in silico* datasets from sortChIC H3K4me3 data.

Scores per task for every *in silico* H3K4me3 dataset pre- and post-imputation. *In silico* datasets have 3 read depth per cell levels (high - 10,000 reads/cell, mid - 1,000 reads/cell, and low 100 reads per cell) and 12 noise levels. Noise levels were grouped into none, high, mid, low and extremely low. The median SiP score over H3K4me3 peaks was shown for (A) all scenarios, and (B) grouped per read depth per cell. The SiP score over the promoters of cell type-specific genes was shown for (C) all scenarios, and (D) grouped per read depth per cell.

Supplementary Figure S4. Comparison of computational approaches for single-cell imputation algorithms on simulations from sortChIC H3K4me3 data.

(A) Final algorithm score across scenarios in algorithms grouped across computational approaches. Kruskal-Wallis test was carried out to calculate variation significance. (B) Final algorithm score across scenarios in algorithms grouped according to whether they were designed for scRNA or scATAC data. A Wilcoxon test was performed to calculate variation significance.

Supplementary Figure S5. Cell-type specific effects on Signal Enrichment after imputation on simulations from sortChIC H3K4me3 data.

(A) Difference in signal enrichment per cell type pre- and post-imputation over 1000 randomly selected H3K4me3 peaks ± 0.5 Mb in datasets with 1000 reads/cell and 1X noise, plotted against the number of cells per cell type. Different colours indicate the specific cell type. (B) Difference in signal enrichment pre- and post-imputation over those same peaks in the previous *in silico* dataset, and a balanced *in silico* dataset from the same scenario (89 cells per cell type). Within each algorithm, the difference in distribution of signal enrichment between the balanced and unbalanced datasets was tested through a Kolmogórov-Smirnov test.

Supplementary Figure S6. Scores per task for the *in silico* datasets from sortChIC H3K4me1, H3K9me3 and H3K27me3 data.

Scores per task for every *in silico* H3K4me1 (A-C), H3K9me3 (D-F), and H3K27me3 (G-I) datasets pre- and post-imputation. *In silico* datasets have 3 read depth per cell levels (high - 10,000 reads/cell, mid - 1,000 reads/cell, and low 100 reads per cell) and 12 noise levels. Noise levels were grouped into none, high, mid, low and extremely low. Plots show the median Pearson correlation to the ground truth (A,D,G), median SIMIC score (B,E,H) and median SiP score over the corresponding peak calls (C,F,I).

Supplementary Figure S7. Summarised scores per task for the *in silico* datasets from sortChIC H3K4me1, H3K9me3 and H3K27me3 data.
Scores per task for every *in silico* H3K4me1 (A-C), H3K9me3 (D-F), and H3K27me3 (G-I) datasets pre- and post-imputation grouped by read depth per cell levels (high - 10,000 reads/cell, mid - 1,000 reads/cell, and low 100 reads per cell. Plots show the median Pearson correlation to the ground truth (A,D,G), median SIMIC score (B,E,H) and median SiP score over the corresponding peak calls (C,F,I).

Supplementary Figure S8. *In silico* comparison of imputation algorithms across epigenetic marks with no added baseline noise or low added noise
Ranking of the imputation algorithms across *in silico* datasets generated from bone marrow sortChIC datasets for H3K4me3, H3K4me1, H3K9me3 and H3K27me3 at mid coverage per cell and (A) no added noise or (C) 0.75X noise. The algorithms are ordered from top to bottom performers according to their median rank across epigenetic marks. (B) Task-specific scores per algorithm per mark for mid-coverage per cell and no added noise.

Supplementary Figure S9. Comparison of single-cell imputation algorithms on simulations from sortChIC H3K4me1, H3K9me3 and H3K27me3 data.
(A), Final algorithm score across scenarios in algorithms grouped across computational approaches. Kruskal-Wallis test was carried out to calculate variation significance. (B) Final algorithm score across scenarios in algorithms grouped according to whether they were designed for scRNA or scATAC data. A Wilcoxon test was performed to calculate variation significance.

Supplementary Figure S10. UMAP visualisations of the datasets after imputation on *in silico* datasets with no added noise across different epigenetic marks.
UMAPs from mid read depth (1000 read/cell) and no added noise for H3K4me3, H3K4me1, H3K27me3 and H3K9me3.

Supplementary Figure S11. Single-cell profiles of all methods across epigenetic marks.
Signal of all imputation algorithms for mid-coverage and 1X noise in Gbe1 locus for H3K4me1 (A) and H3K9me3 (B), and in the Ebf1 locus for H3K27me3 (C).

Supplementary Figure S12. Further evaluation of the performance of single-cell imputation on scCUT&Tag data across several epigenetic marks.
(A) Signal specificity of all methods in a cell type-specific manner for H3K27me3. Signal specificity is represented as the difference in log2 of signal enrichment in intra-cluster peaks (peaks for the corresponding cell type) vs inter-cluster peaks (peaks for all the other cell types). (B) Optimized UMAP representation of all methods on H3K27ac, H3K27me3, H3K36me3 and H3K4me3. Colours denote original cell types. (C) SIMIC scores and improvement of SIMIC scores, compared to no imputation. (D) Confusion-matrices of H3K36me3, H3K27ac, H3K27me3 and H3K4me3 based on K=10 nearest neighbours prediction.

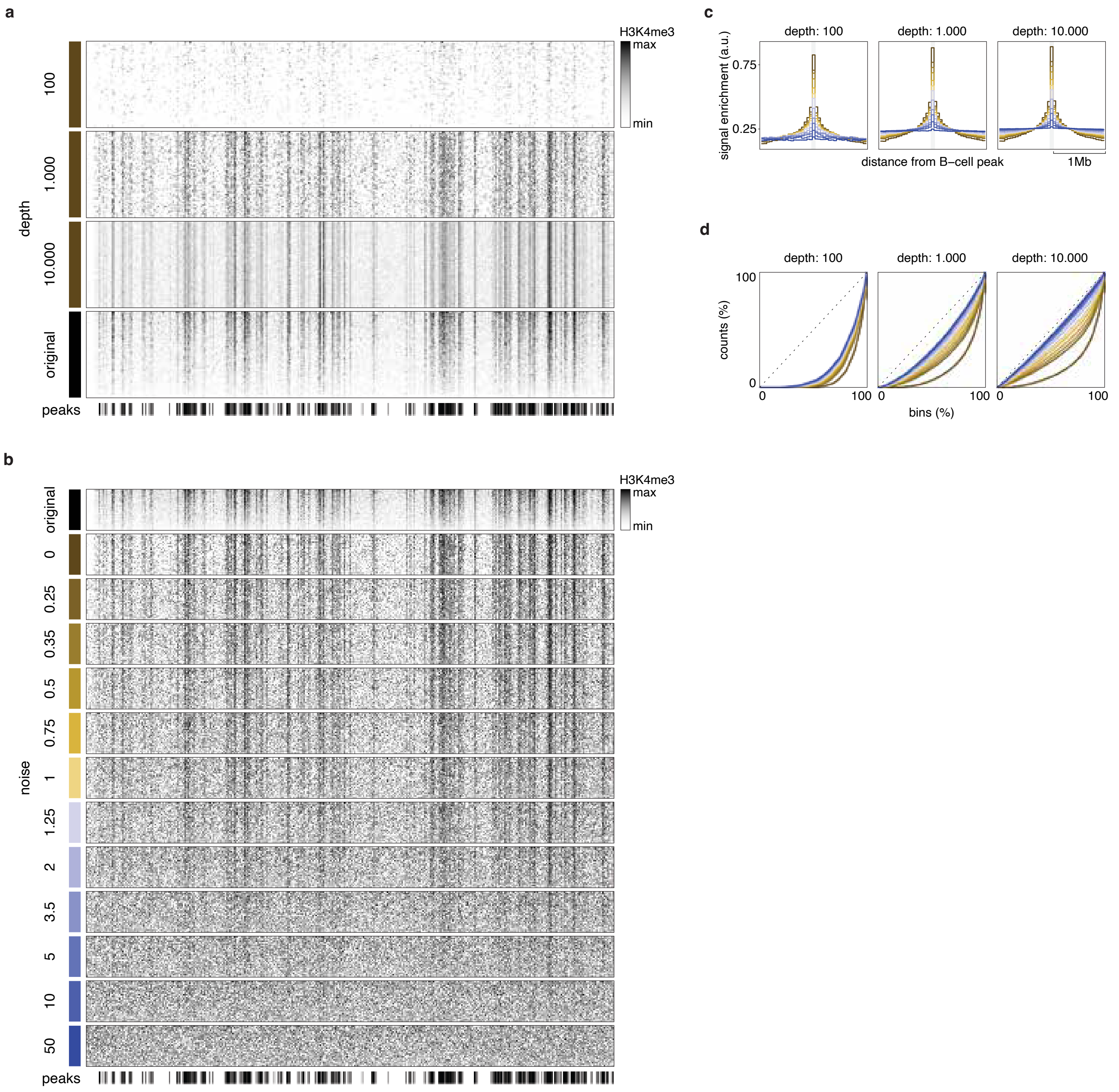

Supplementary Figure 1: Generating *in silico* datasets from H3K4me3 sortChIC data

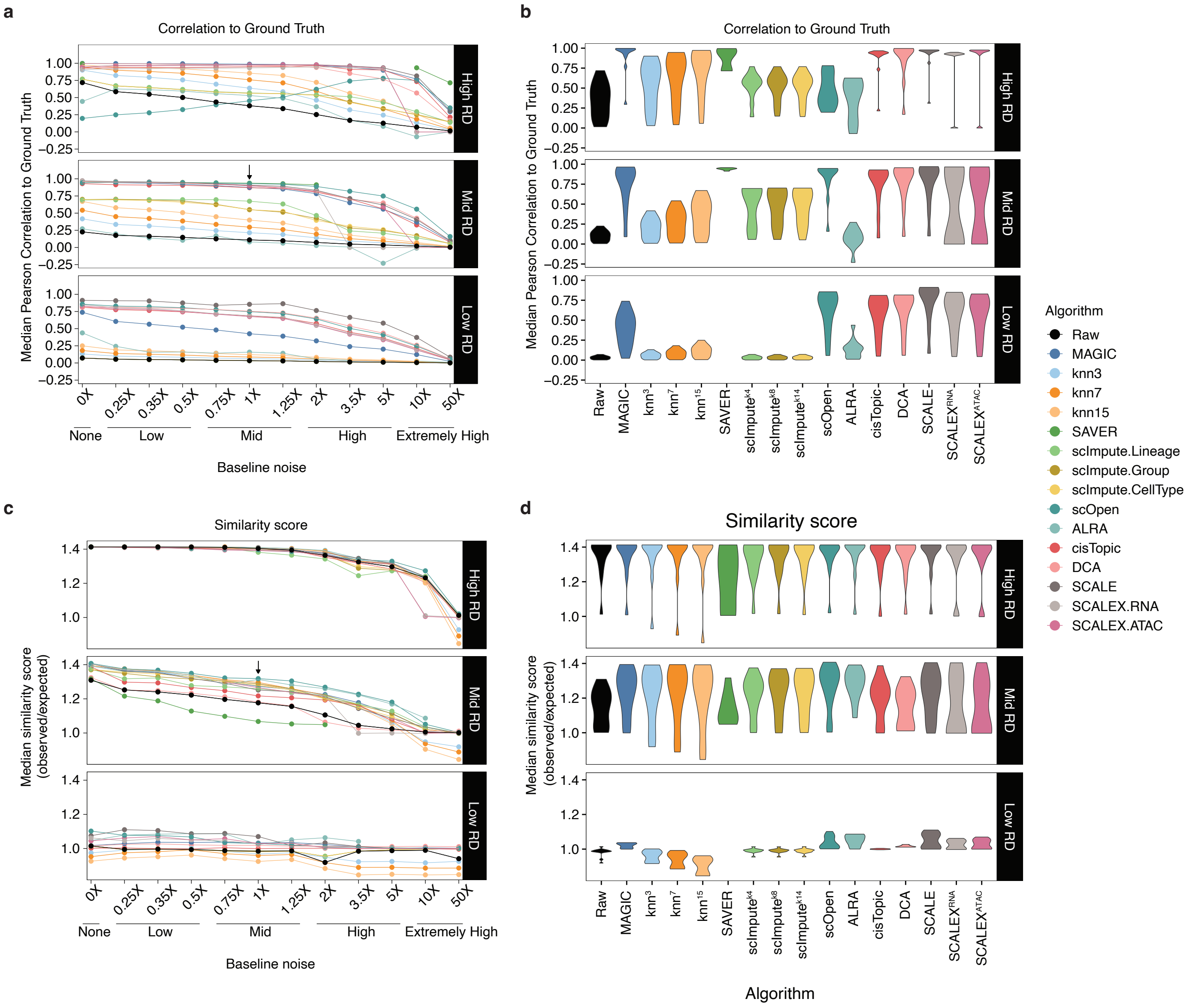

Supplementary Figure 2: Correlation to Ground Truth and similarity scores for the *in silico* datasets from sortChIC H3K4me3 data.

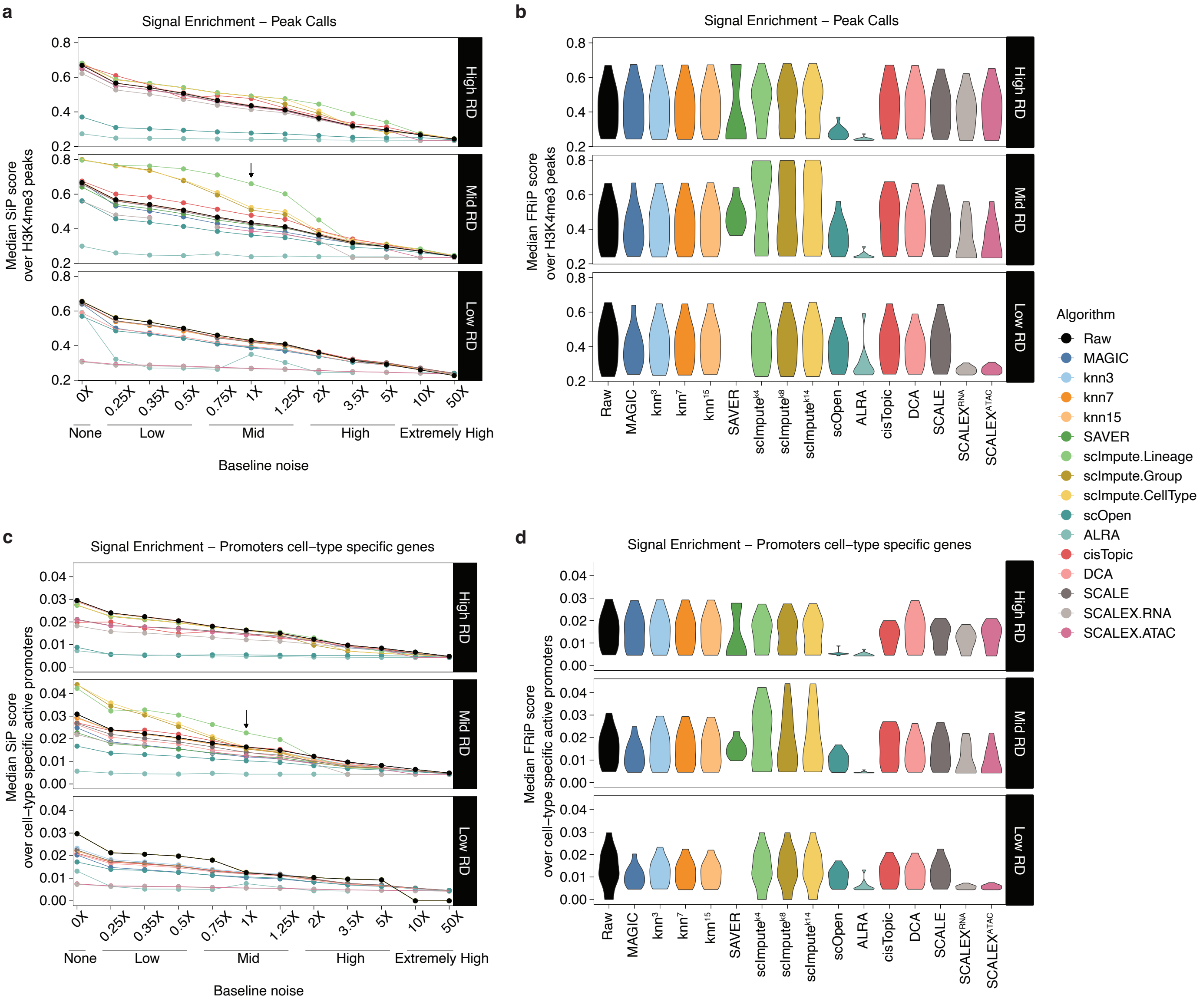

Supplementary Figure 3: Scores for Signal Enrichment for the *in silico* datasets from sortChIC H3K4me3 data.

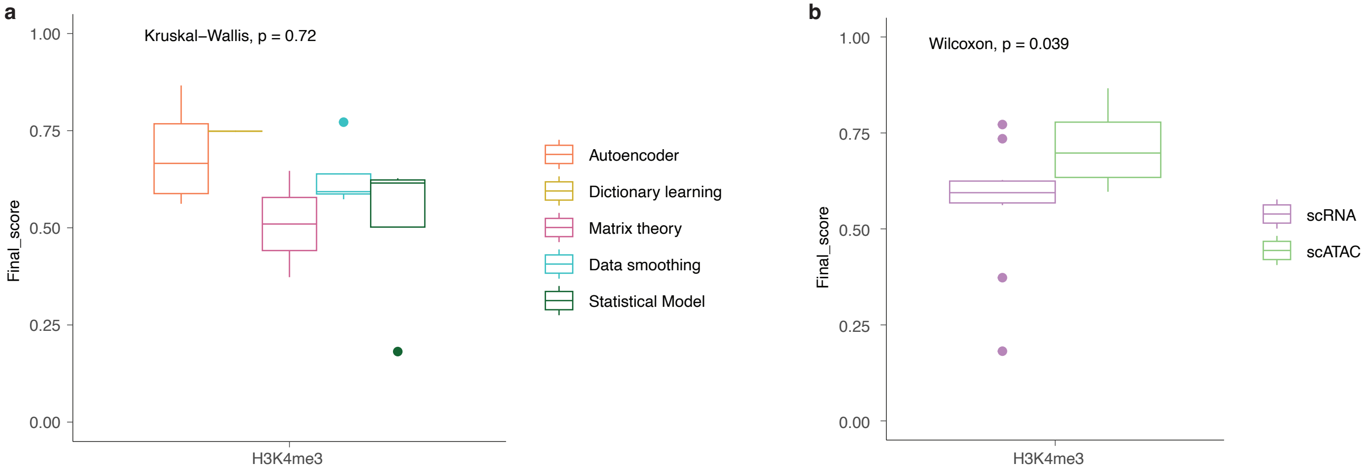

Supplementary Figure 4: Comparison of computational approaches for single-cell imputation algorithms on simulations from sortChIC H3K4me3 data.

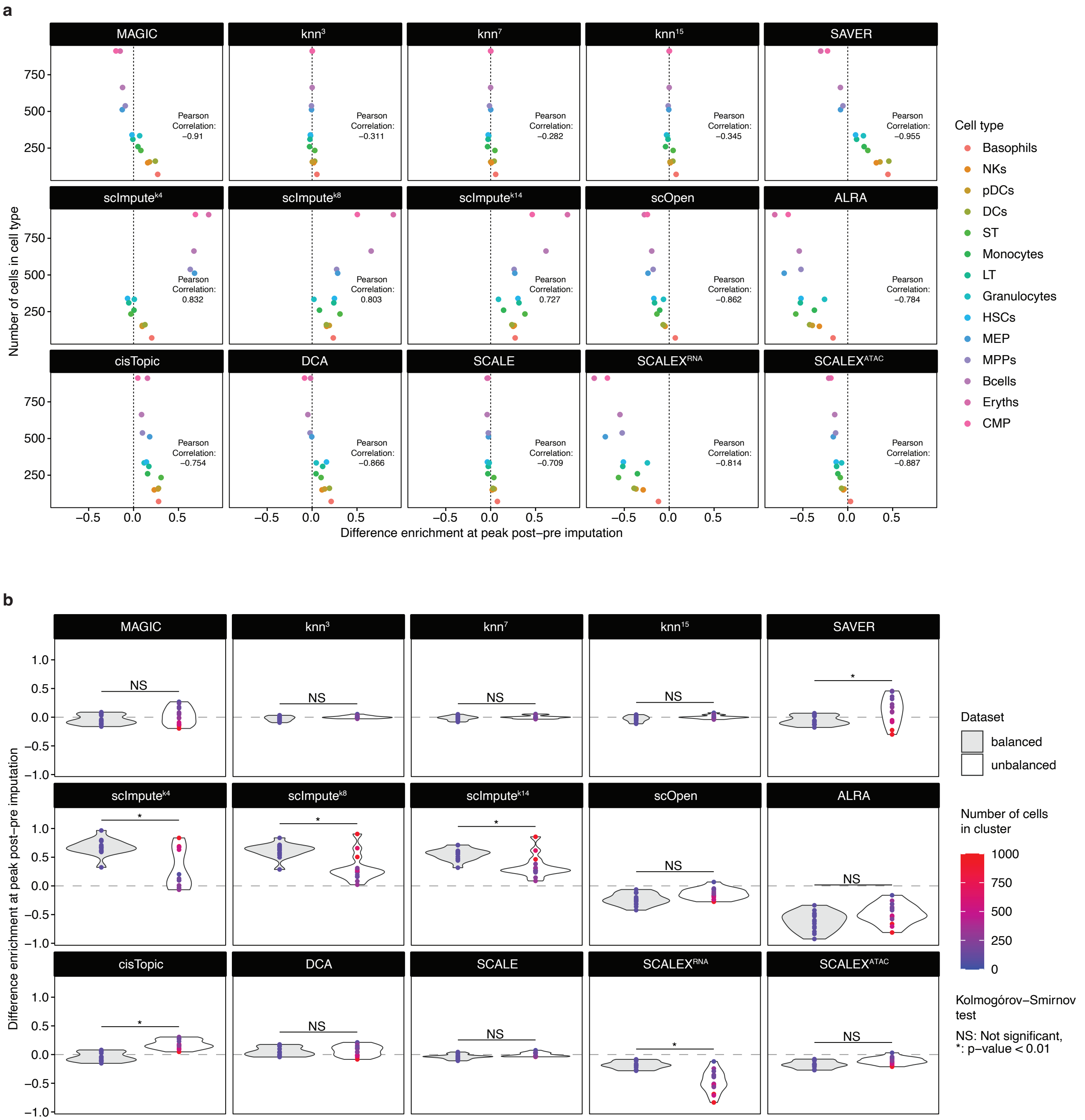

Supplementary Figure 5: Cell-type specific effects on Signal Enrichment after imputation on simulations from sortChIC H3K4me3 data.

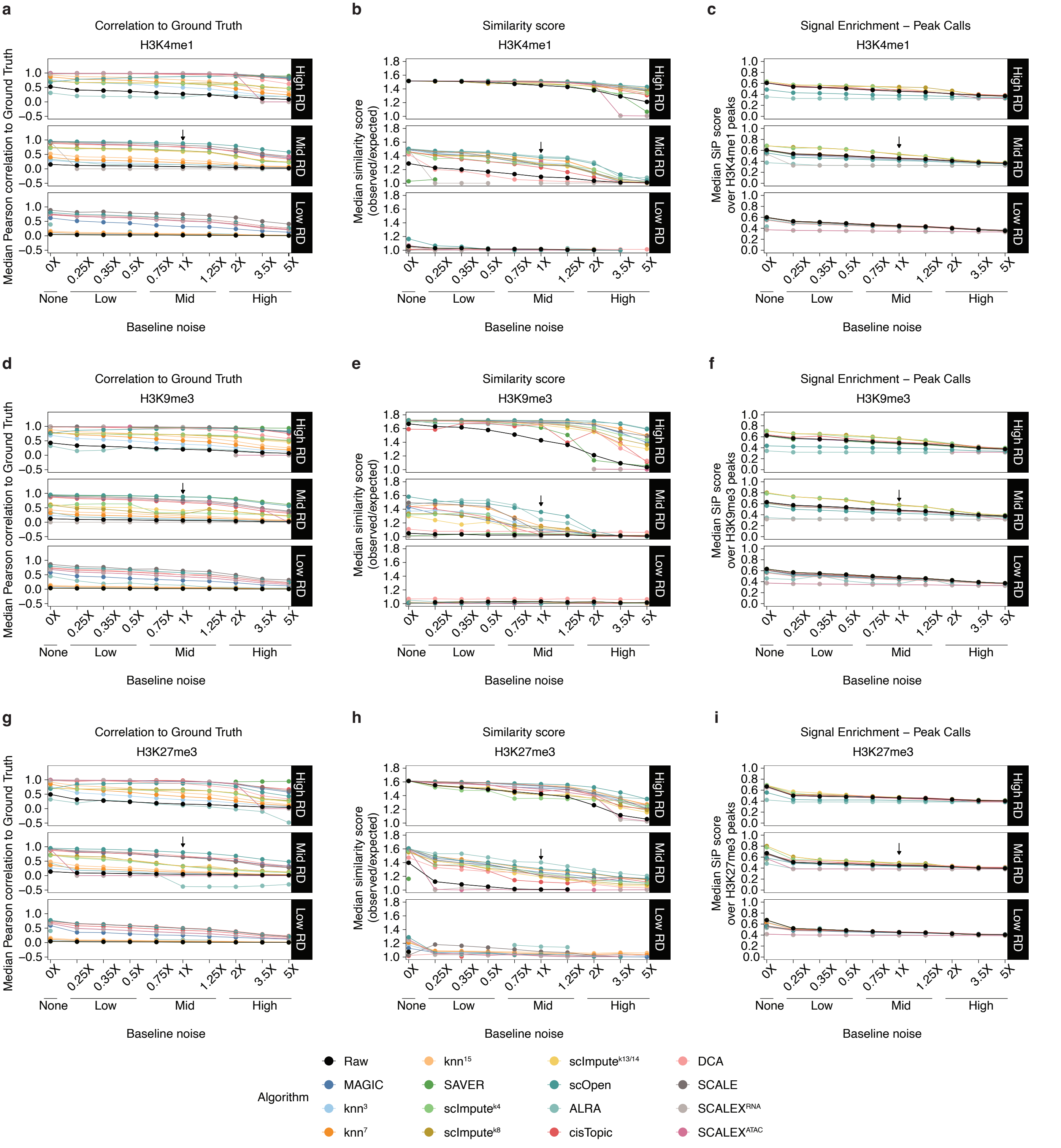

Supplementary Figure 6: Scores per task for the in silico datasets from sortChIC H3K4me1, H3K9me3 and H3K27me3 data

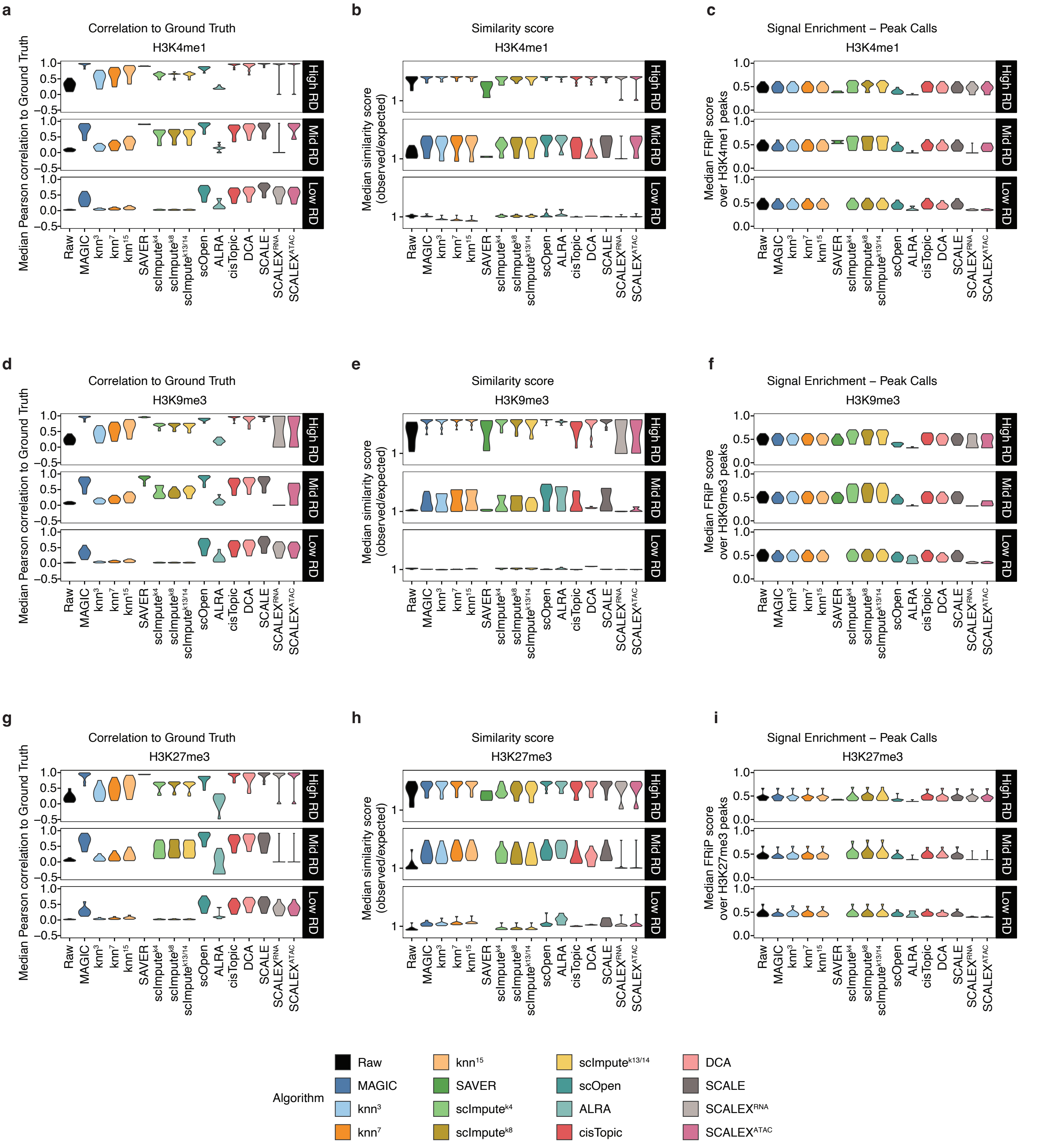

Supplementary Figure 7: Violin plots of the scores per task for the in silico datasets from sortChIC H3K4me1, H3K9me3 and H3K27me3 data

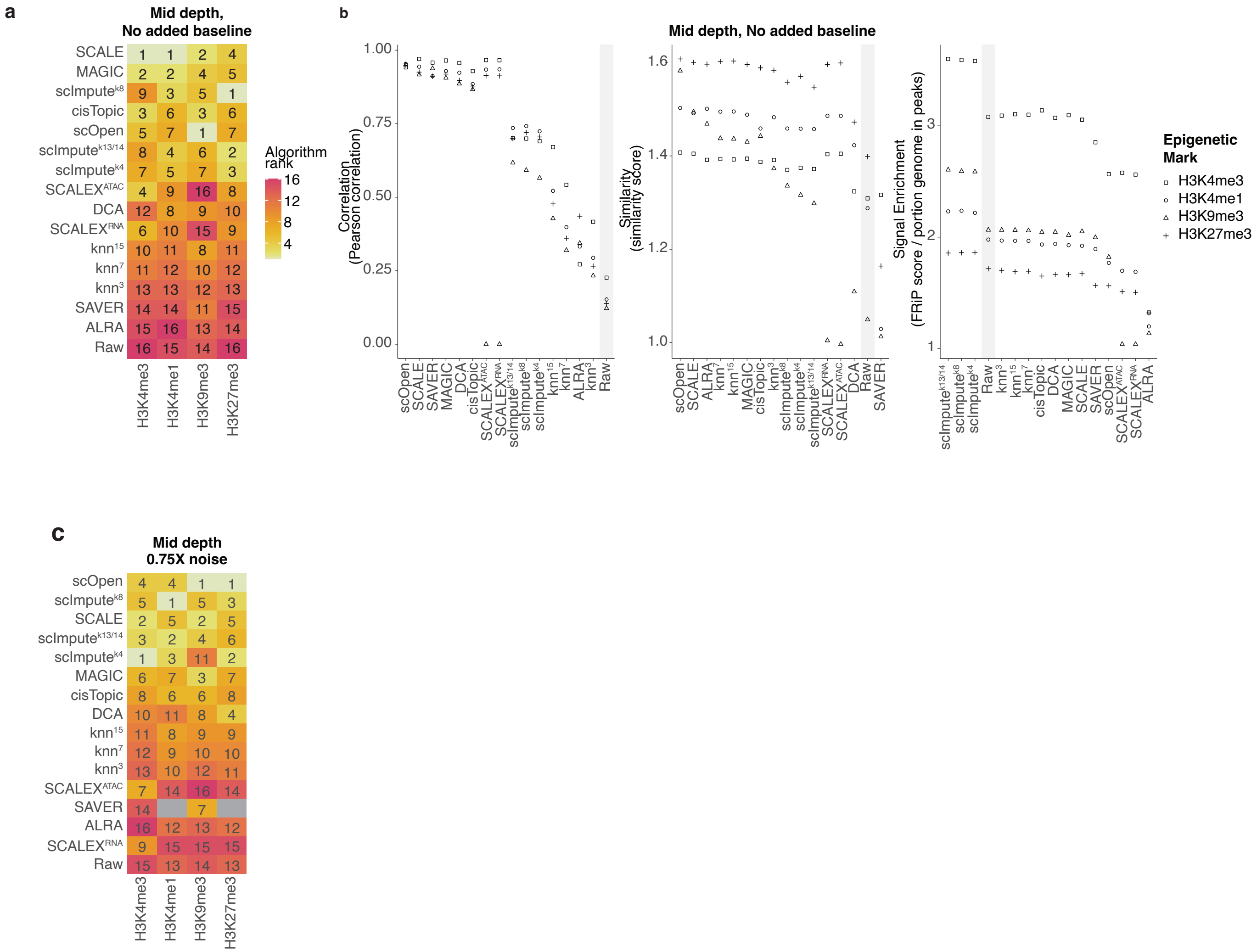

Supplementary Figure 8: ***In silico*** **comparison of imputation algorithms with no added baseline noise or low added noise.**

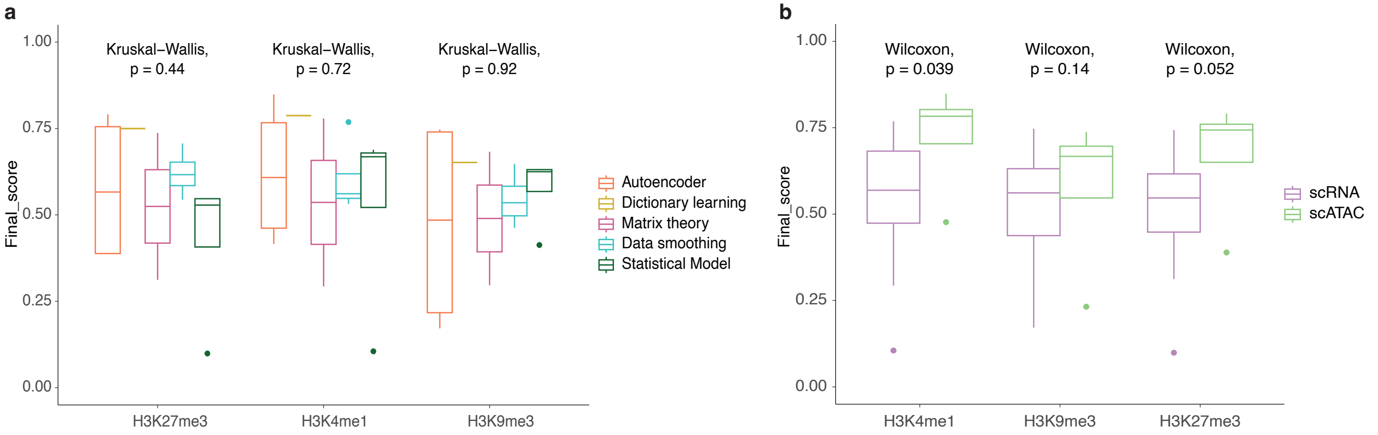

Supplementary Figure 9: Comparison of computational approaches for single-cell imputation algorithms on simulations from sortChIC H3K4me1, H3K9me3 and H3K27me3 data.

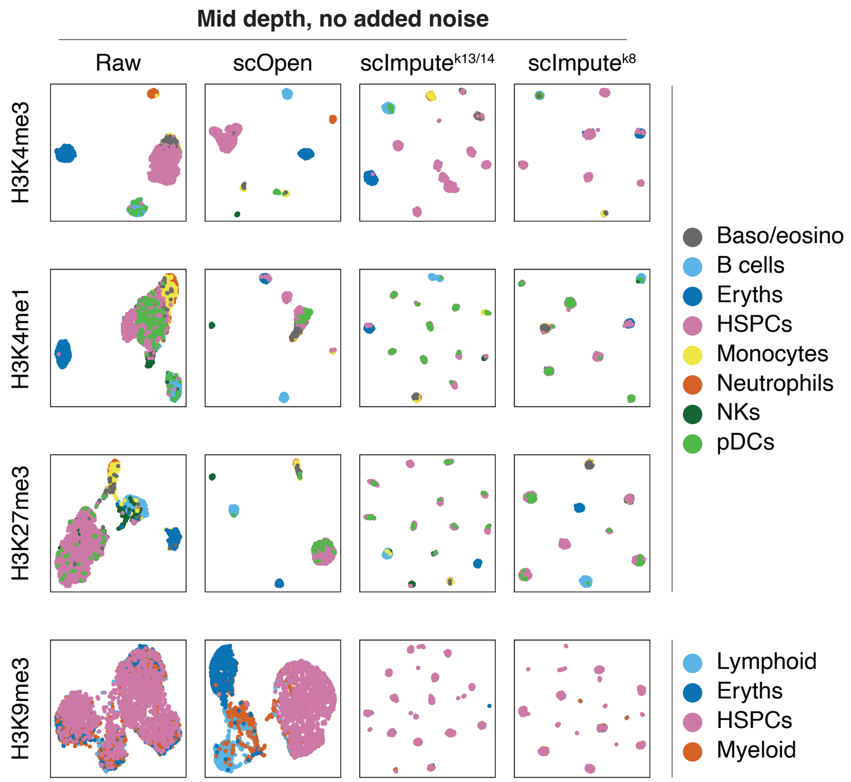

Supplementary Figure 10: UMAP visualisations of the datasets after imputation on *in silico* datasets with no added noise across different epigenetic marks

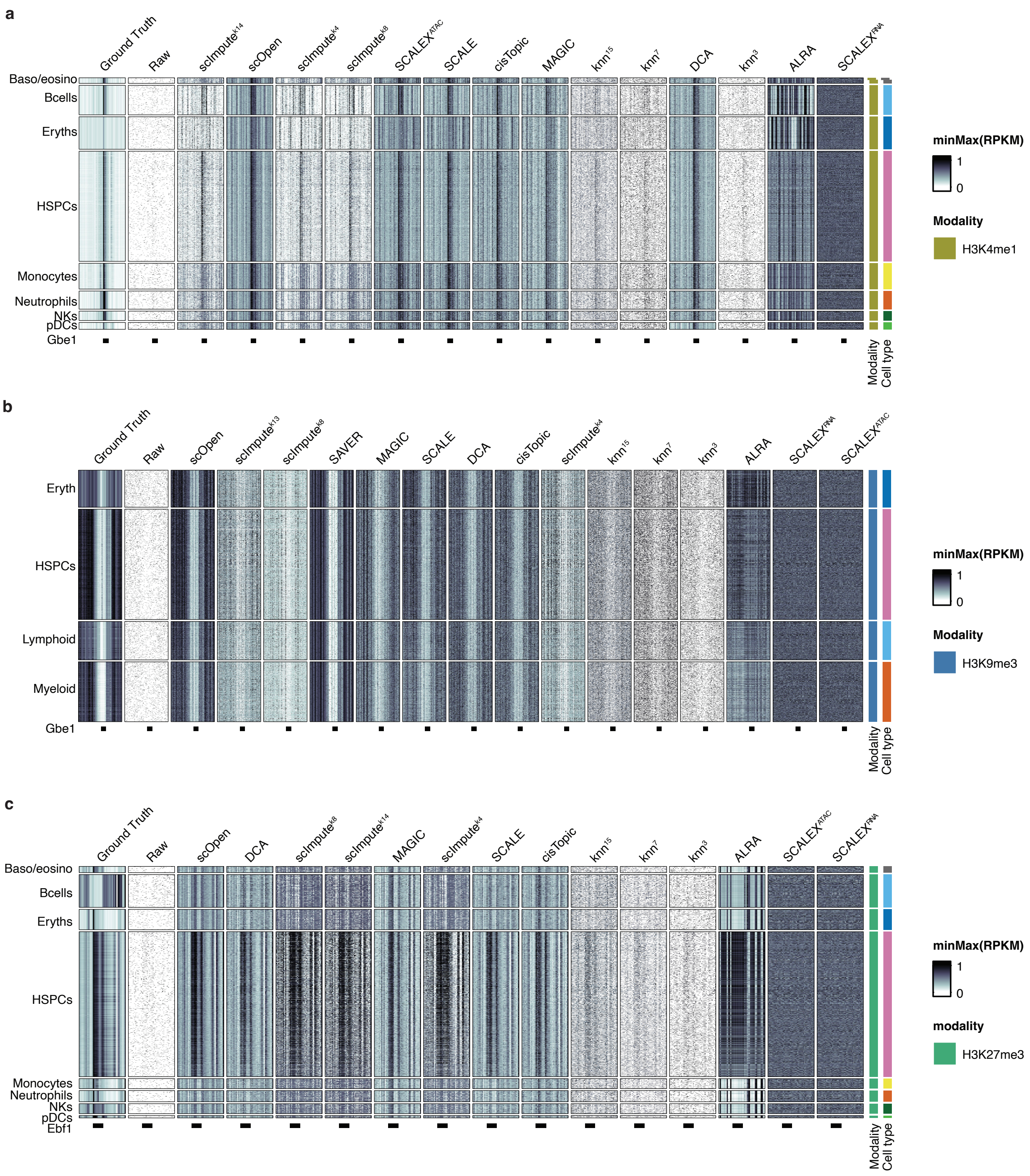

Supplementary Figure 11: Single-cell profiles of all methods across epigenetic marks

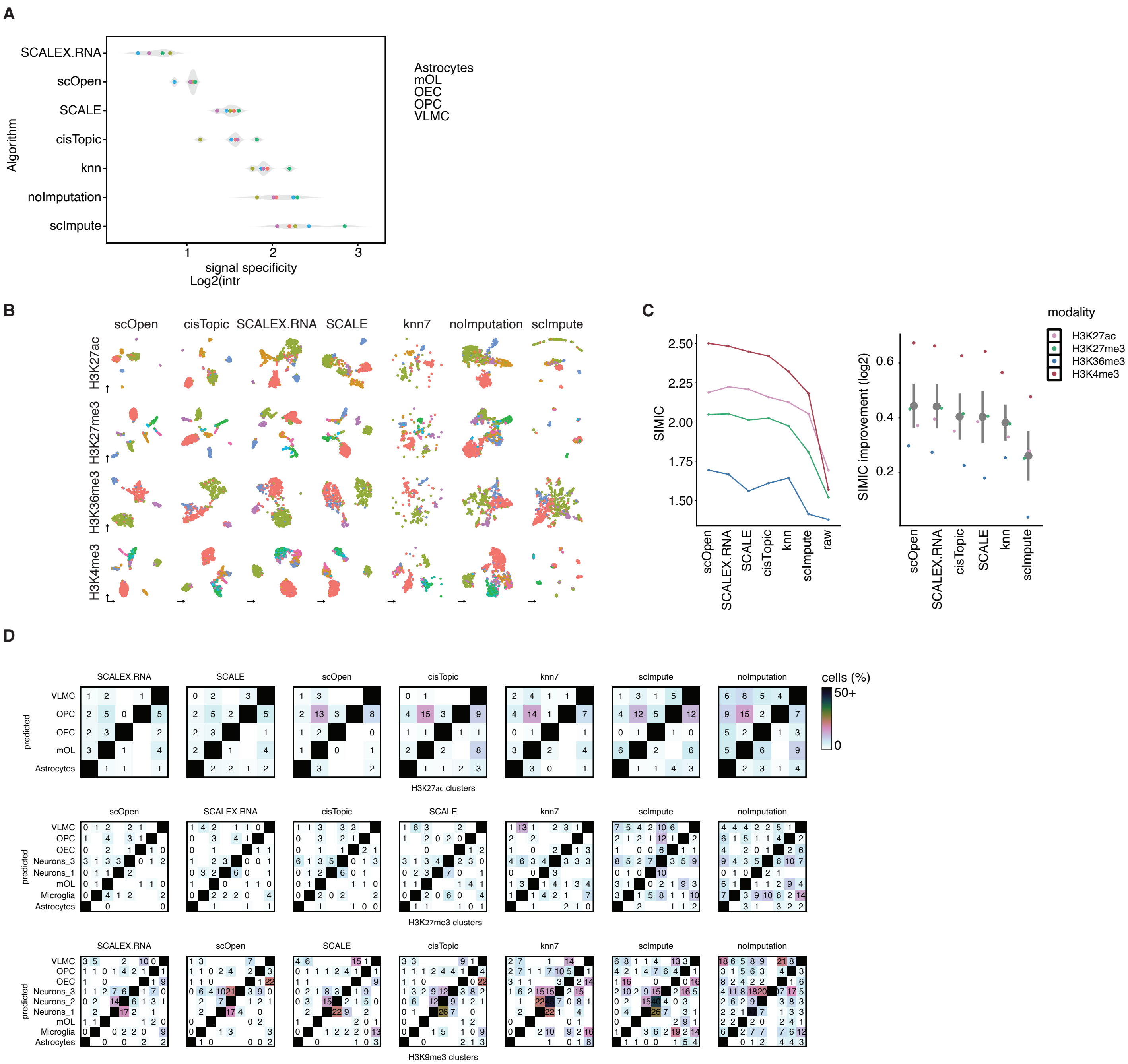

Supplementary Figure 12: Further evaluation of the performance of single-cell imputation on scCUT&Tag data across several epigenetic marks.
